# Dual Task Effects on Visual Attention Capacity in Normal Aging

**DOI:** 10.3389/fpsyg.2018.01564

**Published:** 2018-09-03

**Authors:** Erika C. S. Künstler, Melanie D. Penning, Natan Napiórkowski, Carsten M. Klingner, Otto W. Witte, Hermann J. Müller, Peter Bublak, Kathrin Finke

**Affiliations:** ^1^Hans Berger Department of Neurology, Jena University Hospital, Jena, Germany; ^2^Department of Psychology, Ludwig-Maximilians-Universität München, Munich, Germany

**Keywords:** visual attention, healthy aging, dual-tasking, theory of visual attention, multi-tasking

## Abstract

Older adults show higher dual task performance decrements than younger adults. While this is assumed to be related to attentional capacity reductions, the precise affected functions are not specified. Such specification is, however, possible based on the “theory of visual attention” (TVA) which allows for modeling of distinct attentional capacity parameters. Furthermore, it is unclear whether older adults show qualitatively different attentional effects or whether they show the same effects as younger adults experience under more challenging conditions. By varying the complexity of the secondary task, it is possible to address this question. In our study, participants performed a verbal whole report of briefly presented letter arrays. TVA-based fitting of report performance delivered parameters of visual threshold *t*_0_, processing speed *C*, and visual short-term memory (VSTM) storage capacity *K*. Furthermore, participants performed a concurrent motor task consisting of continuous tapping of a (simple or complex) sequence. Both TVA and tapping tasks were performed under single and dual task conditions. Two groups of 30 younger adults each performed either the simple or complex tapping, and a group of 30 older adults performed the simple tapping condition. In older participants, VSTM storage capacity declined under dual task conditions. While no such effect was found in younger subjects performing the simple tapping sequence under dual task conditions, the younger group performing the complex tapping task under dual task conditions also showed a significant VSTM capacity reduction. Generally, no significant effect on other TVA parameters or on tapping accuracy was found. Comparable goodness-of-fit measures were obtained for the TVA modeling data in single and dual tasks, indicating that tasks were executed in a qualitatively similar, continuous manner, although quantitatively less efficiently under dual- compared to single-task conditions. Taken together, our results show that the age-specific effects of motor-cognitive dual task interference are reflected by a stronger decline of VSTM storage capacity. They support an interpretation of VSTM as central attentional capacity, which is shared across visual uptake and concurrent motor performance. Capacity limits are reached earlier, and already under lower motor task complexity, in older compared to younger adults.

## Introduction

Aging is associated with a decline of sensory and motor functions, as well as distinct cognitive abilities (Lindenberger, 2014). Moreover, consistent evidence shows that dealing with cognitive demands in parallel to a motor task is more difficult for subjects of a higher age (McDowd and Craik, 1988; Kramer and Larish, 1996; Verhaeghen and Cerella, 2002; Woollacott and Shumway-Cook, 2002; Verhaeghen, 2011; Ruthruff and Lien, 2017). Thus, not only do cognitive and motor skills both decline over the life span (Ketcham and Stélmach, 2001; Park and Reuter-Lorenz, 2009; McAvinue et al., 2012; Habekost et al., 2013), but dual tasking seems to add an additional deteriorating factor (Verhaeghen et al., 2002, 2003) that renders even the execution of seemingly easy tasks vulnerable through the introduction of a secondary task (Boisgontier et al., 2013; Künstler et al., 2017). That is, dual tasking requirements seem to represent a specific challenge for elderly adults, which in turn leads to exacerbated performance deterioration. These particular difficulties of older adults in dual tasking situations are especially relevant because they have been linked to a higher risk of falls (Faulkner et al., 2007). However, the reasons for these stronger dual task effects in aging are still not entirely clear.

Dual task interference is observed when performance of one or both tasks within a dual task situation declines compared to the performance of each single task carried out separately (Kahneman, 1973). Two of the most influential attentional explanations for the dual task effect are the bottleneck account and the central capacity sharing model (see Tombu and Jolicoeur, 2004, for an overview). According to the bottleneck account, the dual task related decline in performance arises from the fact that two tasks cannot be executed simultaneously but have to be carried out in a sequential manner, at least at some stage of processing (Pashler, 1994). In contrast, the capacity sharing account assumes simultaneous task performance, but suggests that the overall amount of attentional resources available for performance is strictly limited (e.g., Navon and Miller, 2002). Due to this limitation, attentional capacity has to be shared between the two tasks, giving rise to a trade-off in task performance. As long as the individual's capacity limit is not reached, both tasks can be performed concurrently without a drop-off in either task. Only when the task demand exceeds said limit, one or both of the tasks will be affected. Capacity sharing models consider serial task processing at central stages (Pashler, 1994) as a special case of capacity sharing, whereby first Task 1 and then Task 2 gets all of the available capacity. However, Logan and Gordon (2001) offered a model combining aspects from both the resource sharing and the bottleneck account in their “executive control of the theory of visual attention” (ECTVA) framework.

The “theory of visual attention” (TVA; Bundesen, 1990; see Bundesen et al., 2015 for a current overview) can itself be applied as a framework to assess processing capacity under a dual task condition. TVA is a mathematically formalized theory which has strong relations to the biased competition account of attentional processing. With the Neural Theory of Visual Attention (NTVA) Bundesen et al. (2005) sought to describe single cell data based on TVA, thereby attempting to provide a deeper understanding of how TVA could possibly be explained from a neural standpoint. TVA disentangles processing capacity into a set of distinct parameters determining the efficacy of an individual's visual information uptake. These parameters can be estimated by modeling participants' performance on a simple psychophysical whole report task (e.g., Sperling, 1960). In this task, an array of letter stimuli is briefly presented; TVA proposes that these stimuli are encoded in two distinct processing waves. The first, unselective wave processes the visual information in parallel, allocating evidence values to objects based on the extent to which long-term memory representations match the objects in the display. The second, selective wave distributes limited capacity attention across the objects, with attentional weighting being allocated based on the evidence values. The objects then race to be encoded in the fixed capacity visual short-term memory, which is typically limited to approximately three to four elements in younger, healthy participants. This VSTM storage capacity is intimately related to the concept of visual working memory capacity, as applied by Luck and Vogel (2013) and proposed to be a central index of overall cognitive ability (however, see Aben et al., 2012 for an opposing view). Only those objects which are encoded into the VSTM store are consciously represented, and are therefore available for further actions, such as verbal report.

Performance in the whole report task is modeled, according to the equations set out by TVA (see Kyllingsbaek, 2006; Habekost, 2015, for a comprehensive overview), by an exponential growth function that relates accuracy of letter report to the effective stimulus exposure duration. The origin, the slope, and the asymptote of this function are determined by three parameter estimates provided by TVA: the perceptual threshold, *t*_0_, reflects the time-point at which conscious visual stimulus processing starts; the processing rate *C* indexes the number of visual elements which can be processed per second; and parameter *K* estimates the size of the storage capacity of the visual short-term memory, given as the maximum number of elements which can be maintained in parallel. TVA has several advantages in the dual tasking context (see Habekost, 2015, for an overview on the methodological merits of TVA-based measurement): Importantly, to the best of our knowledge, TVA-based testing furthermore is the only methodology that permits a mathematically *independent quantification* measurement of the parameters perceptual threshold, processing speed, and capacity of VSTM. Thus, firstly, it reveals cognitively specific information on which aspect(s) of visual attentional processing is or are affected by the concurrent second task. Secondly, it allows precise measurements of how strongly each parameter is affected. Furthermore, as the TVA whole report paradigm does not rely on motor speed or button presses, the effects of a concurrent manual motor task can be assessed simultaneously, without motor confounds. Finally, by analyzing goodness of fit parameters, qualitative comparisons between single- and dual-task performance can be made, giving insights into how the tasks are processed.

In a recent study Künstler et al. (2017) assessed motor-cognitive dual task interference by combining the TVA-based whole report task with a simple motor task (alternating tapping with two fingers of the dominant hand) in middle- to higher-aged individuals. The results revealed a decline of visual attentional capacity under dual task conditions. Importantly, goodness-of-fit and reliability measures in both single and dual task conditions showed that participants performed on the visual task in a qualitatively similar (i.e., continuous), although quantitatively less efficient way under dual task as compared to single task conditions. Taken together, the results supported a capacity sharing account of motor-cognitive dual-tasking and suggested that even performing a relatively simple motor task relies on central attentional capacity that is necessary for efficient visual information uptake.

In the present study, we apply this method to analyse the effects of aging on motor-cognitive dual-task performance. We investigate which attentional capacity aspects are disproportionately affected in older compared to younger adults when performing a concurrent motor task consisting of the continuous tapping of a simple sequence. In an additional group of younger participants, the complexity of the tapping sequence was increased. This was done due to the evidence that older subjects require more attention for the execution of simple motor tasks, which younger subjects can perform more or less effortlessly (Boisgontier et al., 2013). That is, we tested the hypothesis that more pronounced effects in the older group are attributable to the motor demand being more challenging for them. Taken together, by quantifying the dual-task decrement in older and younger adults, we firstly want to specify the exact attentional parameters that are more prone to dual-task decline in older compared to younger adults. Secondly, by comparing the dual-task decrements of older adults induced by a simple tapping sequence to the decline induced by a more complex sequence in younger adults, we want to assess whether older adults show the same dual-task effects as younger adults facing a more challenging dual-task scenario.

## Methods

This study combined a TVA whole report paradigm with a simple or complex continuous tapping task as the secondary task. In order to establish the effect of task load, 30 younger participants completed a simple tapping task condition (referred to as the “younger simple group”), while 30 younger adults performed a more complex tapping sequence as the secondary task (the “younger complex group”). Then, to look at the effects of aging, the performance of the 30 younger adults who executed the simple tapping sequence was compared to the performance of 30 older adults who completed the same task (the “older adults group”). This allowed us to explore the decline in dual-task abilities as a function of age. Lastly, to test whether younger participants experience a qualitatively similar decline in attentional processing under more complex conditions, we compared the performance of the older adults to that of the younger adults who completed the complex tapping task.

### Participants

We tested a total of 90 participants, split into three groups of 30 participants each, who were recruited at the Department of Psychology, Ludwig Maximilians Universität, in Munich and the Department of Neurology, Jena University Hospital, in Jena, Germany: An older group aged between 50 and 78 years, one younger group aged between 19–35 years performing a simple tapping sequence and another younger group with an age of 18–34 years performing a complex tapping sequence. All participants had normal or corrected to normal vision and no history of neurological or psychiatric disorders. The older participants were tested for signs of beginning dementia (MMSE; all values ≥ 27; all values ≥ 26; and MOCA; Folstein et al., 1975; Nasreddine et al., 2005). Handedness was assessed with the Edinburgh Handedness Inventory (Oldfield, 1971) and vocabulary as an estimate of crystallized intelligence with the “Mehrfachwahl-Wortschatz-Test” (MWT-B; Lehrl, 1977). Due to changes in educational and occupational standards over the years, we created a sociodemographic score based on vocabulary (an estimate of crystallized intelligence), number of school years, and occupation (please see the Supplementary Material for a full overview of how this score was constructed). This sociodemographic score indicated that there were no significant differences between the various groups. The study was approved by the Ethics Committees of the Jena University Hospital and of the Ludwig-Maximilians-Universität München, and all participants gave written informed consent prior to participation, in accordance with the Declaration of Helsinki. Each participant received monetary remuneration. Relevant demographic data for each group are listed in Table 1.

**Table 1:** Demographic data and sociodemographic score for younger participants who performed the simple or complex tapping sequence and for older participants who performed the simple tapping sequence.

**Variable**	**Older (*N* = 30)**	**Younger simple (*N* = 30)**	**Younger complex (*N* = 30)**
Gender (N): m/f	16/14	18/12	13/17
Handedness: r/a	29/1	30/0	30/0
Age (years): Mn/SD/range	65.0/7.6/50–78	26.1/3.8/19–35	25.7/4.1/18–34
Sociodemographic score: Mn/SD/range	7.4/1.3/5–9	6.7/1.4/4–9	7.2/1.1/5–9

*Demographics include gender (number), handedness (number), age, and sociodemographic score*.

*M, male; f, female; r, right; a, ambidextrous; Mn, Mean; SD, standard deviation*.

### Apparatus

In both locations, the data was collected in dimly lit- and sound-attenuated rooms so as to minimize distractions. Stimuli were presented on ASUS VG248 17-inch monitors with a refresh rate of 100 Hz and a resolution of 1920 × 1080 and a viewing distance of 60 cm. The tapping task was conducted on external keyboards attached to the computer on which the experiments were run. The height of the screen was adjusted for each participant, such that the center of the screen was directly at eye level. Because of the setup of the apparatus, the keyboard was located below participants' visual periphery. Thus, to visually monitor their tapping performance, participants would have had to move their heads downwards so as to see their hands. Not only were participants instructed to not look down, and to continuously maintain fixation at the center of the screen, but their compliance was also monitored by the examiner.

### Procedure

All participants completed a single session which lasted around 60 min. Approximately 20 min were spent on questionnaires aimed at obtaining demographic information. The remaining 40 min were allocated to the tapping tasks and TVA based whole report, with breaks being taken as needed. The task order was counterbalanced between participants, such that half of all participants began with the two single tasks before commencing to the dual-task condition, while the other half started with the dual-task condition, before completing the two single tasks. In this case, the single tapping was always first performed first.

#### Tapping task

**Figure 1:**
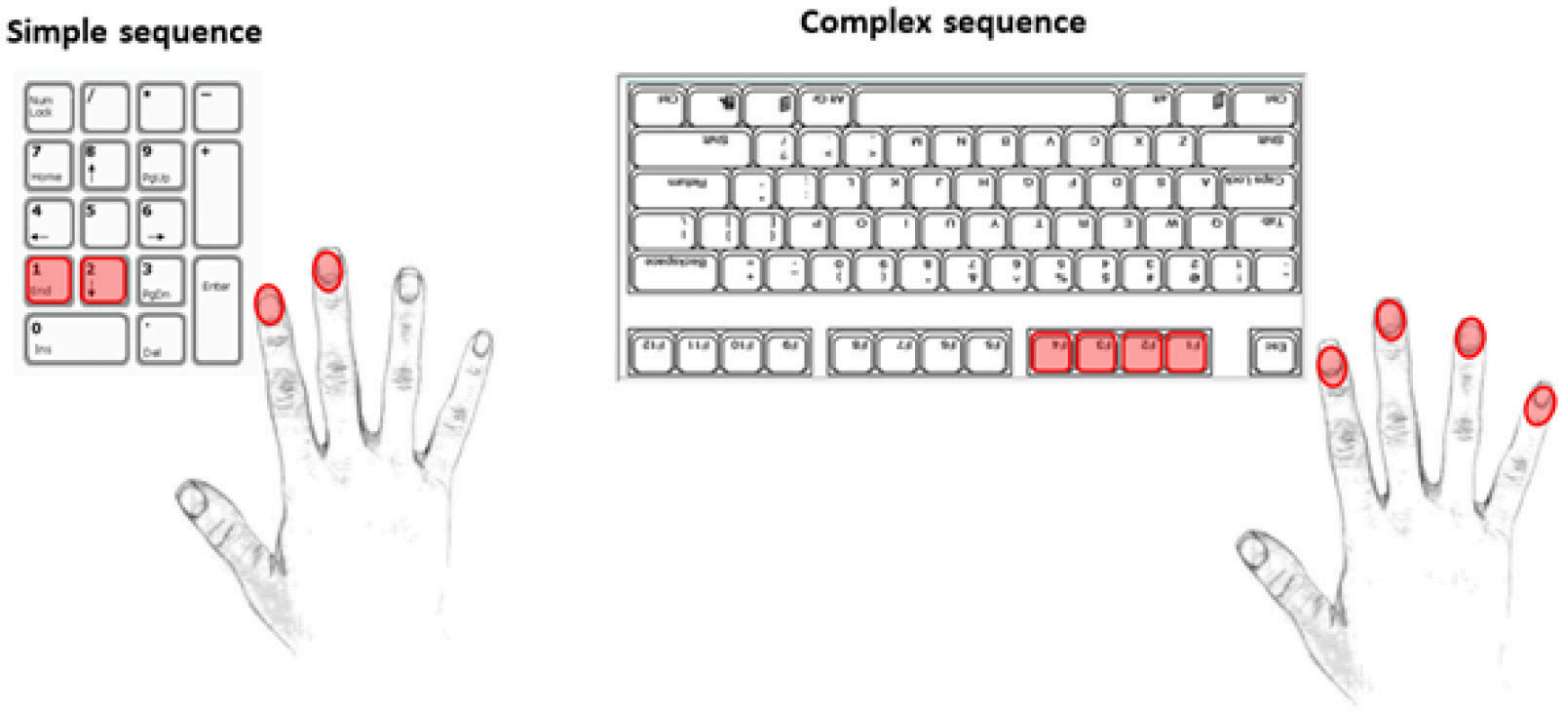
Simple and complex tapping sequences. Used keys and fingers are marked in red.

This task was carried out using the dominant hand to continuously tap a given sequence. The simple sequence consisted of using the index and middle fingers to press the “1” and “2” keys respectively, while the more complex sequence required the use of the index, middle, ring, and pinky fingers to press the “F4,” “F3,” “F2,” and “F1” keys (with the keyboard turned upside down to reduce interference from other keys) respectively (see Figure 1 for a diagrammatic representation of these two sequences). The more complex sequence was deduced from an unpublished pilot study in which we tested the effects of varying sequence complexities in younger participants. The complex sequence used in the current study was found to be moderately challenging, but manageable for most participants.

The allocated sequence was then tapped at a subjectively preferred pace for a prespecified amount of time. As per the methodology used by Kane and Engle (2000), the single condition of the tapping task consisted of three blocks. The first block spanned 30 s, and was used to familiarize the participant with the sequence to be tapped. If performance on this block was unsatisfactory, the block could be repeated. However, if the performance on the first block was above 80% accuracy, the participant could go on to the second block, which lasted 60 s, during which time the average tapping speed was calculated. In this block, if the wrong key was pressed, auditory feedback in the form of a beep was given to the participant. If this block was performed below 80% accuracy, it could be repeated. However, if performance was satisfactory, the participant could proceed to the third block. Here, the average tapping speed calculated in the second block was added to a buffer of 150 ms. This was then used as the cut-off speed for the third block. Thus, if a participant took longer than this cut-off speed to press a key, or if the wrong key was pressed, a beep was again used as auditory feedback. This final block lasted 3 min, as this time-frame is equivalent to the average duration of a block in the whole report task. It was also a reasonable duration which should not lead to discomfort or hand cramps for the participants according to experience from a previous study (Künstler et al., 2017). A text file was created which recorded the time stamps and tapping speed for each key press, along with information about which key was pressed. This information allowed the *post-hoc* calculation of each participant's speed and accuracy, and also allowed the time-stamps to be compared between tasks in the dual-tasking condition. The average tapping accuracy and standard deviations for all groups and conditions can be found in Table 2,^1^.

**Table 2:** Tapping accuracy and TVA parameter values across all conditions and groups.

**Parameters**	**Older**		**Younger simple**		**Younger complex**	
	**Single Task**	**Dual Task**	**Single Task**	**Dual Task**	**Single Task**	**Dual Task**
Tapping accuracy: Mn/SD/N	97.5/4.6/30	96.4/3.3/29	98.8/1.4/29	98.8/1.2/30	96.2/4.6/29	96.3/3.2/30
WR minimum EDs: Mn/SD/N	12.0/4.8/30	14.0/7.2/30	10.0/0.0/30	10.0/0.0/30	11.0/4.0/30	10.7/3.7/30
WR maximum EDs: Mn/SD/N	202.3/5.0/30	204.3/7.3/30	200.7/2.5/30	200.7/2.5/30	201.7/4.6/30	201.3/4.3/30
Parameter *K:* Mn/SD/N	3.1/0.6/30	2.8/0.6/30	3.7/0.7/30	3.7/0.7/30	3.8/0.8/30	3.5/0.8/30
Parameter *C:* Mn/SD/N	31.7/ 9.2/30	28.6/12.8/30	34.3/16.6/30	31.4/14.2/30	31.2/15.4/30	30.2/14.3/30
Parameter *t0:* Mn/SD/N	11.9/13.5/30	12.4/13.9/30	−1.8/15.1/30	−3.0/ 13.1/30	−1.4/15.2/30	−3.1/15.9/30

*Mn, Mean; SD, standard deviation; N, sample size; WR, Whole Report; ED, exposure duration*.

#### Whole report task

**Figure 2:**
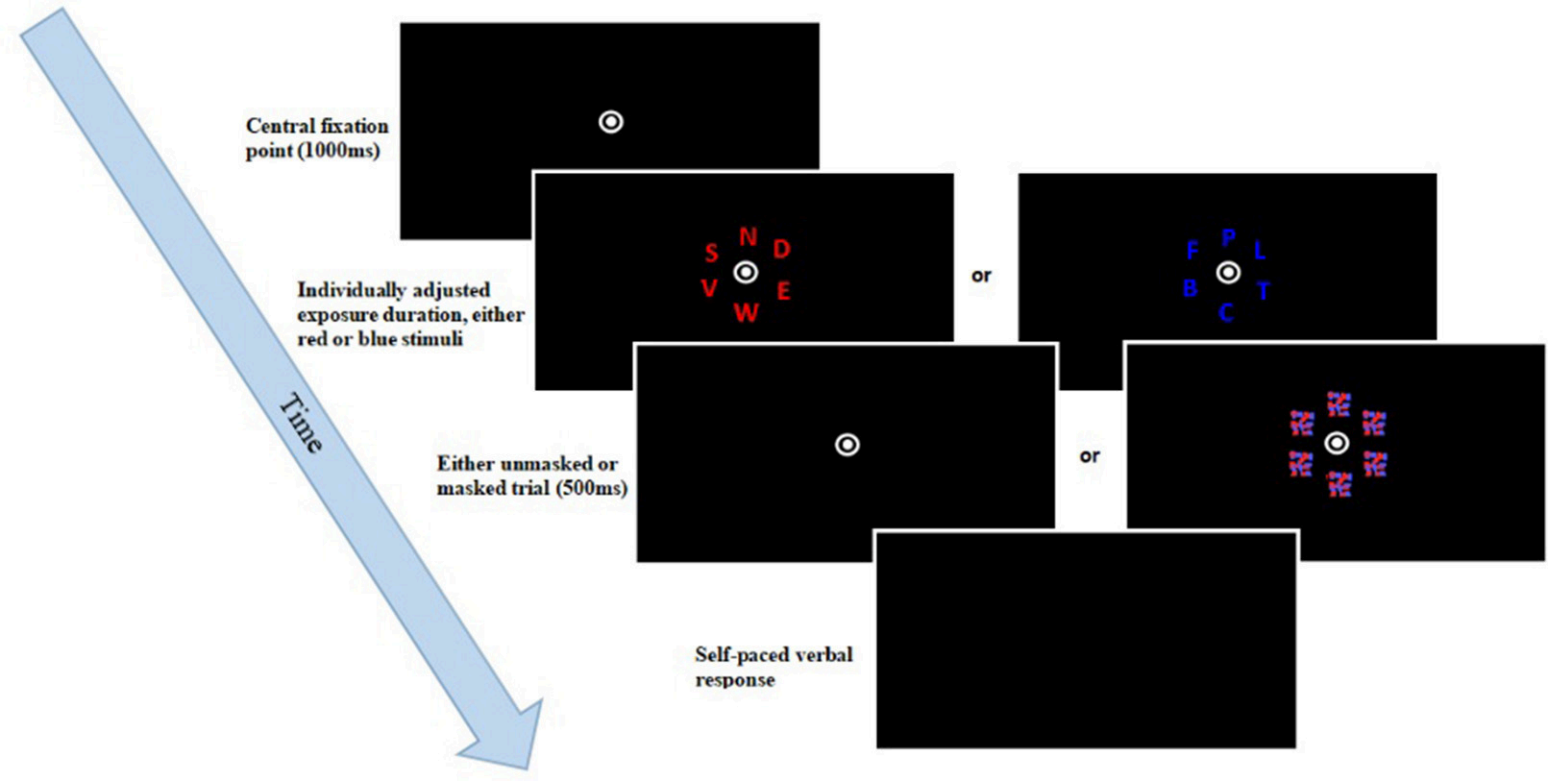
TVA whole report trial sequence. After the presentation of a fixation point, six either red or blue letters were briefly displayed, followed by a mask in some of the trials. Participants had to name all letters they had recognized.

This task was run in Matlab^2^, using Psychtoolbox (Brainard, 1997). The experiment consisted of a total of 140 trials. At the start of each trial, a fixation point was displayed in the center of the screen for 1,000 ms. Subsequently, six isoluminant letters appeared around the fixation point, displayed equidistantly in an invisible circle. These letters were drawn at random from a predefined set of letters (all letters of the alphabet, excluding I, Q, and Y), with the size being set to 1.5 by 1.5 cm. These letters were either all blue [Color space: CIE L × a × b blue = (17.95; 45.15; —67.08)] or red [CIE L × a × b red = (28.51; 46.06; 41.28)], with a luminance of 0.49cd/m2. In 40 trials, the stimuli were masked. Once the screen went blank, participants were tasked with verbally reporting as many of the observed letters as possible; an unspeeded task, thereby allowing each participant as much time as necessary. The responses were then typed in by the researcher, who was seated behind the participant, before going on to the next trial. The timestamps of the responses, as well as the responses made, and the correct responses were exported to a text file. Following each block, participants received accuracy feedback on-screen, indicating what percentage out of the letters actually reported was correct. Performance between 70 and 90% was seen as optimal. If the accuracy rate dropped below 70%, participants were asked to be more conservative in their answers. If their accuracy was above 90%, participants were asked to try reporting more letters. A diagrammatic representation of a trial sequence can be found in Figure 2. The mean accuracy for this criterion in the single and dual task conditions was 87.6 (*SD* = 4.7) and 86.4 (*SD* = 4.2) for the older group, 86.5 (*SD* = 6.6) and 85.8 (*SD* = 6.4) for the younger simple group, and 87.5 (*SD* = 5.8) and 85.1 (*SD* = 5.6) for the younger complex group.

Initially, the task instructions were displayed on-screen, followed by two examples. Subsequently, a pretest, consisting of 12 triples of trials, was run over the course of four blocks. This served to familiarize the participants with the task, as well as to individually adjust the exposure duration to each participant through the use of a Bayesian adaptive staircase model. Two of the trials in each triple were not used for adjustment; one was unmasked with exposure duration of 200 ms, while the other was masked and presented for 250 ms. This long exposure duration was only used to familiarize the participant with the task; in the experiment itself, shorter, and adjusted exposure durations were used. Only one trial in each triple was critical for exposure adjustment; this was masked and initially displayed for 100 ms. If at least one letter in such a critical trial was reported correctly, the exposure duration was decreased by 10 ms in the following critical trial. This was repeated until a final exposure duration was identified at which the participant was unable to report any letter correctly. This was then taken to be the lowest exposure duration, and was used together with four other pre-set exposure durations, which were picked based on the lowest, individually adjusted exposure duration. Stimuli in five conditions, using the different exposure durations, were masked. These masks, which comprised a red/blue mesh of overlapping flecks, were 2 by 2 cm in size, and covered the stimuli for 500 ms. They were used to avoid visual persistence effects, as visual information in unmasked trials typically persists by several hundred milliseconds (Sperling, 1960; Dick, 1974). In addition to these five masked conditions, two unmasked conditions were used, using the second shortest and the longest exposure duration, giving rise to a total of seven effective exposure duration conditions. Such a broad spectrum of exposure durations is necessary to measure a wide range of performance, allowing for the estimation of different parameters. For example, *t*_0_, the perceptual threshold, is calculated based on performance changes at lower exposure durations close to the minimum individual effective exposure duration. Exact quantification of *t*_0_ is in turn needed to determine the rate of information uptake at *t*_0_, indexed by parameter *C*. However, the computation of the VSTM storage capacity, which is demarcated by the asymptote of performance or parameter *K*, requires higher exposure durations. For each of the seven effective exposure conditions, 20 trials were included in the study, resulting in a total of 140 trials, divided into four experimental blocks. The obtained data could then be further analyzed through the LibTVA script (Dyrholm, 2012) in Matlab^2^ which calculated a maximum likelihood fit for the data, according to the principles of TVA. This was done for each participant, and utilizes observed data to extrapolate probabilistic parameters, based on the fixed capacity independent race model (see Shibuya and Bundesen, 1988). Our model had eight degrees of freedom: Five for parameter *K* and one each for parameters *C, t0*, and μ (“iconic memory buffer,” of no particular interest to this study). The average minimum and maximum exposure durations for each group and condition can be found in Table 2.

#### Dual-task

In this condition, participants completed the whole report task while simultaneously and continuously tapping. Participants initially performed the familiarization and speed adjustment blocks of the tapping task, after which the whole report paradigm was started. This was then followed by the simultaneous execution of both tasks concurrently, while participants' gaze remained fixated to the center of the screen. The timestamps of the data points of both tasks were compared. If the participant made a mistake in the tapping task, then the corresponding trial in the whole report task was excluded from the analysis. This was done in order to examine attentional parameters only in those trials where the tapping was successfully executed. On average, 5.7 (*SD* = 6.9) trials were excluded in the older simple group, 3.1 (*SD* = 4.3) trials were excluded in the younger simple group and 9.0 (*SD* = 7.2) trials were excluded in the younger complex group. Supplementary Table 4 shows how the exclusion of trials affected Goodness-of-Fit values.

### Goodness of fit

As the whole report results were obtained through a mathematical model, we wanted to ensure that the observed data was closely mirrored by the estimated parameters. To this end, we did a Goodness of Fit analysis. These Goodness of Fit values give an indication of how much of the variance of the empirically observed data is explained by the model estimates provided by TVA. Thus, the higher the explained variance, the more closely the parameter estimates match the actual data obtained.

Furthermore, these Goodness of Fit results also provided an estimation of how robust these estimates were between the single and dual task conditions. More precisely, TVA posits that the processes indexed by the parameter estimates remain stable across comparable conditions. Violations of this assumption, e.g., due to the switching between tasks, would be expected to result in a lower Goodness of Fit in the dual task condition.

## Results

The accuracy of the letter whole report was modeled as a function of effective exposure duration for each participant and task condition (single whole report task condition, dual task condition), from which parameters *K* (VSTM storage capacity in number of objects), *C* (visual processing speed in objects/s) and *t*_0_^3^ (visual threshold in ms) were derived. For the tapping task, overall accuracy was computed for each task condition (single tapping task condition, dual task condition). The means and standard deviations of these parameter estimates are given for each group in Table 2.

We computed separate repeated-measures ANOVAs for tapping accuracy and TVA parameters. For comparison of older participants performing the simple tapping sequence to either younger participants performing the simple tapping sequence or younger participants performing the complex tapping sequence we included the factors Age Group (older vs. younger) and Task Condition (single task vs. dual task). Three tapping accuracy values were missing (one from each group) due to technical errors. For the sake of interest, several further analyses can be found in the Supplementary Materials, including a comparison between the two younger groups. Furthermore, for individual values of TVA parameters and tapping accuracy see Supplementary Table 4, while the individual variability in TVA parameter *K* is provided in Supplementary Figures 2–4.

### Older group performing the simple tapping sequence vs. younger group performing the simple tapping sequence

To look for age effects on tapping accuracy and TVA parameters in a dual task situation a comparison was run between older and younger participants who both performed the simple tapping sequence.

#### Tapping

**Figure 3:**
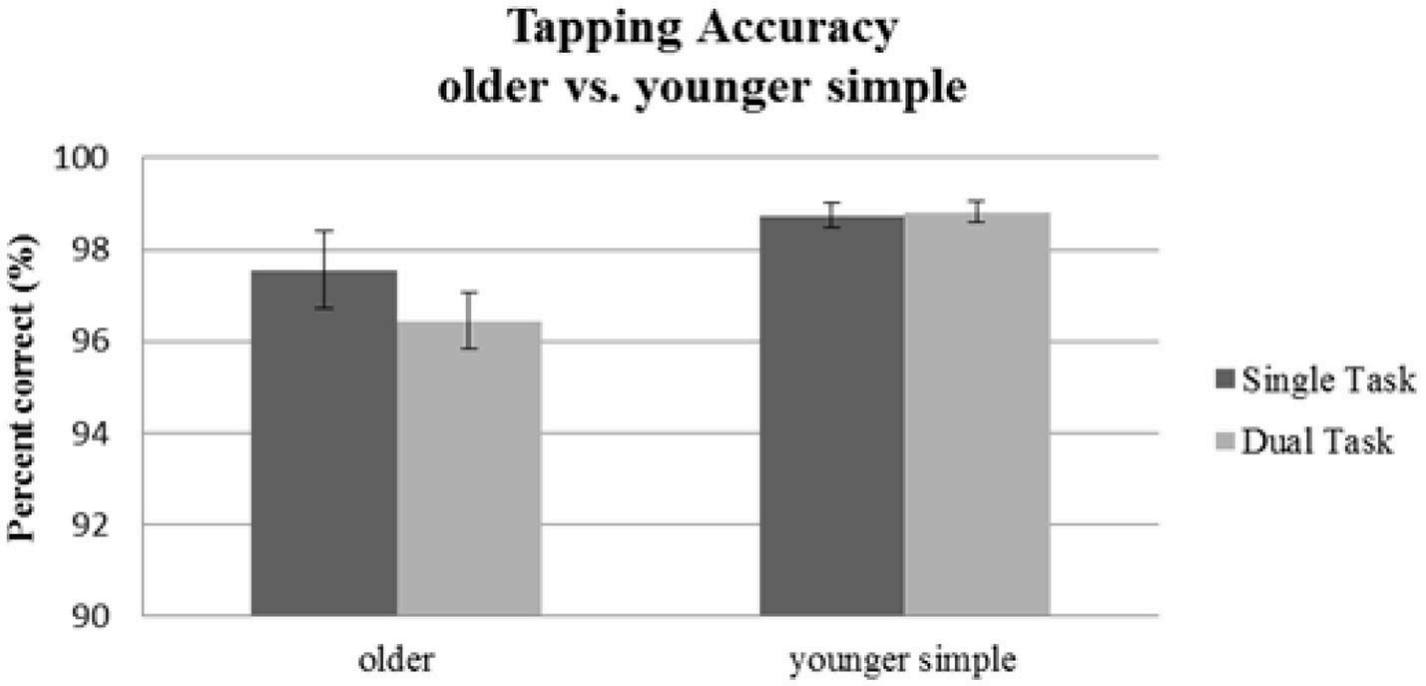
Tapping accuracy as indicated by percentage of correct taps for the older group performing the simple tapping sequence vs. the younger group performing the simple tapping sequence. Error bars indicate standard errors of the mean.

For tapping accuracy (see Table 2), we found a significant main effect of Age Group [*F*_(1, 56)_ = 7.06, *p* = 0.01; $\eta_{p}^{2}$ = 0.11]. The main effect of Task Condition [*F*_(1, 56)_ = 1.56, *p* = 0.22; $\eta_{p}^{2}$ = 0.03], and the interaction [*F*_(1, 56)_ = 2.06, *p* = 0.16; $\eta_{p}^{2}$ = 0.04] were not significant. Thus, younger and older participants differed in their general tapping accuracy, but neither group's tapping accuracy was affected by the concurrent visual task. Results are depicted in Figure 3.

#### Whole report

**Figure 4:**
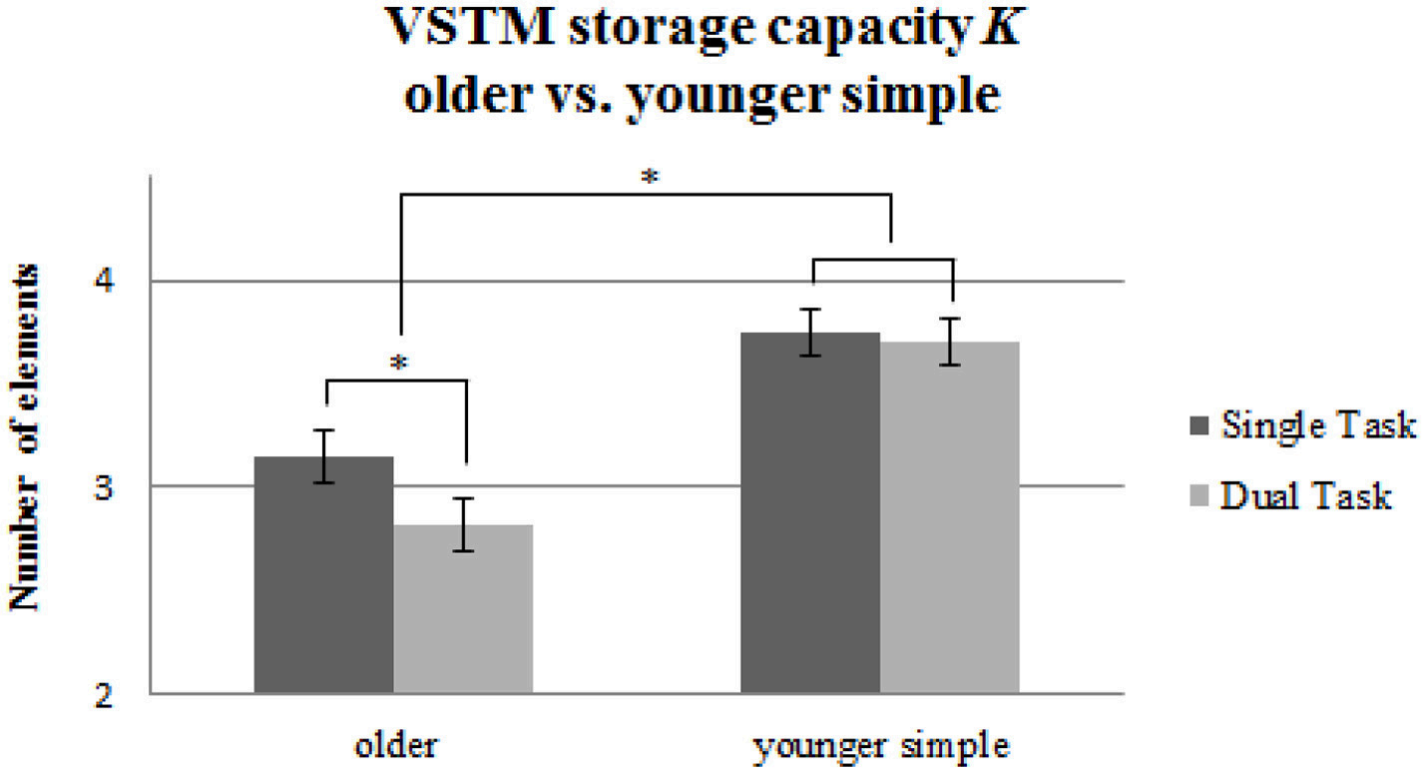
VSTM capacity *K* measured in maximum number of recognized letters for the older group performing the simple tapping sequence vs. the younger group performing the simple tapping sequence. Error bars indicate standard errors of the mean. Significant differences are denoted by an asterisk (^*^).

For VSTM storage capacity *K* (see Table 2), we found significant main effects of Age Group [*F*_(1, 58)_ = 19.91, *p* < 0.001, $\eta_{p}^{2}=$ 0.26] and Task Condition [*F*_(1, 58)_ = 17.05, *p* < 0.001, $\eta_{p}^{2}=$ 0.23], and a significant interaction [*F*_(1, 58)_ = 10.01, *p* = 0.002, $\eta_{p}^{2}=$ 0.15; see Figure 4]. *Post-hoc* pairwise *t*-tests with Bonferroni-correction demonstrated that there was a significant decline in VSTM storage capacity in the older group induced by the tapping [*t*_(29)_ = 4.49, *p* < 0.001, *d* = 0.52], while, as described before, the younger group performing the same, simple tapping sequence did not show this effect [*t*_(29)_ = 0.83, *p* = 0.41, *d* = 0.06].

For processing speed *C* (see Table 2) no significant main effect of Age Group was found [*F*_(1, 58)_ = 0.76, *p* = 0.39; $\eta_{p}^{2}=$ 0.01]. There was a trend for an effect for Task Condition, indicating lower performance in the dual-task compared to the single-task condition across groups [*F*_(1, 58)_ = 3.37, *p* = 0.07; $\eta_{p}^{2}=$ 0.06]. The interaction was not significant [*F*_(1, 58)_ = 0.002, *p* = 0.97; $\eta_{p}^{2}<$ 0.0014]. Thus, there was no indication for a general age effect or for an increased dual task effect with increased age.

Similar effects as for processing speed were also found for the perceptual threshold parameter *t*_0_ (see Table 2). There was only a significant effect for Age Group [*F*_(1, 58)_ = 20.09, *p* < 0.001; $\eta_{p}^{2}=$ 0.26], while the main effect for Task Condition [*F*_(1, 58)_ = 0.06, *p* = 0.81; $\eta_{p}^{2}=$ 0.001] and the interaction [*F*_(1, 58)_ = 0.27, *p* = 0.60; $\eta_{p}^{2}=$ 0.005] were not significant. Thus, significantly higher thresholds for older compared to younger adults were found in both task conditions, while there was no evidence for an age-specific dual task decrement for visual threshold t_0_.

### Older group performing the simple tapping sequence vs. younger group performing the complex tapping sequence

Older participants' performance was also compared to that of the younger participants who completed the complex tapping sequence to see whether younger participants would show comparable effects as older participants under a more challenging dual-task condition.

#### Tapping

**Figure 5:**
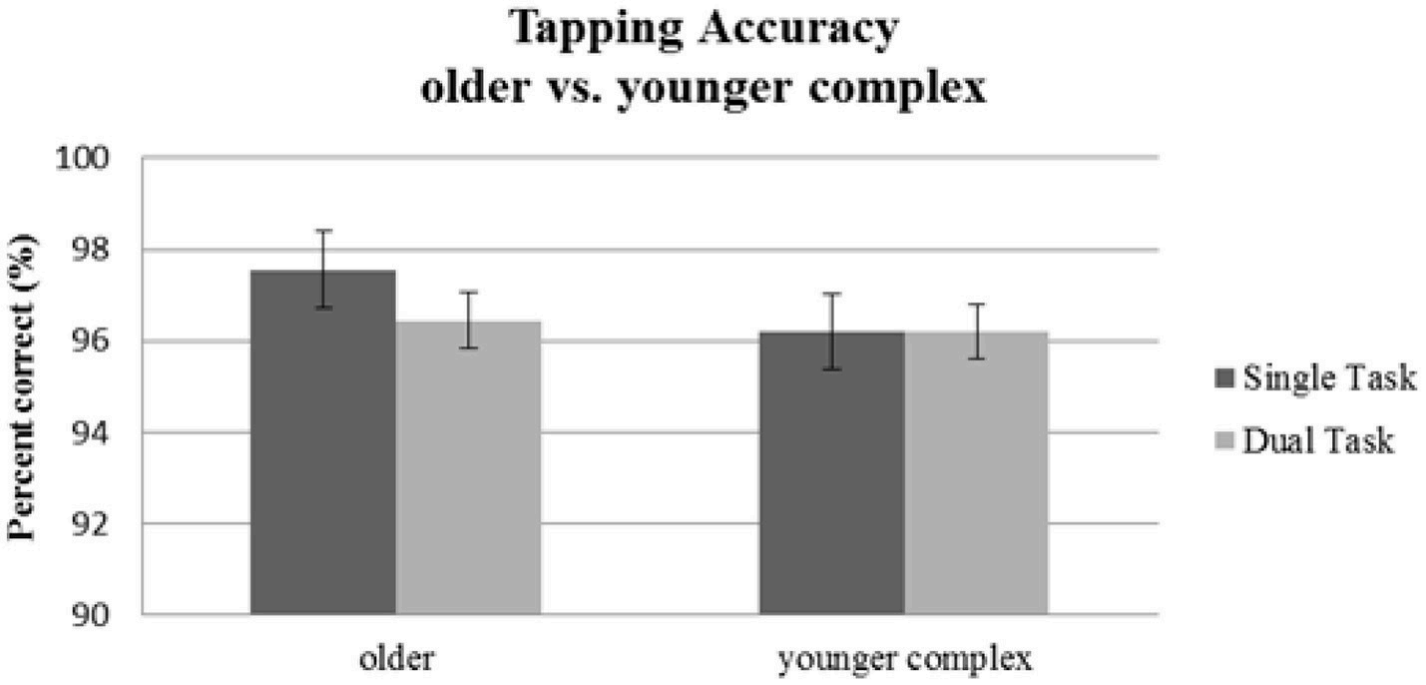
Tapping accuracy as indicated by percentage of correct taps for the older group performing the simple tapping sequence vs. the younger group performing the complex tapping sequence. Error bars indicate standard errors of the mean.

No significant main effect of Age Group [*F*_(1, 56)_ = 0.79, *p* = 0.38; $\eta_{p}^{2}=$ 0.01] or Task Condition [*F*_(1, 56)_ = 0.99, *p* = 0.33; $\eta_{p}^{2}=$ 0.02] was found on tapping performance. The interaction [*F*_(1, 56)_ = 1.05, *p* = 0.31; $\eta_{p}^{2}=$ 0.02] was also not significant. Thus, neither older participants nor younger adults performing a complex tapping sequence showed dual-task effects on motor performance induced by an additional visual attention task (see Table 2, Figure 5).

#### Whole report

**Figure 6:**
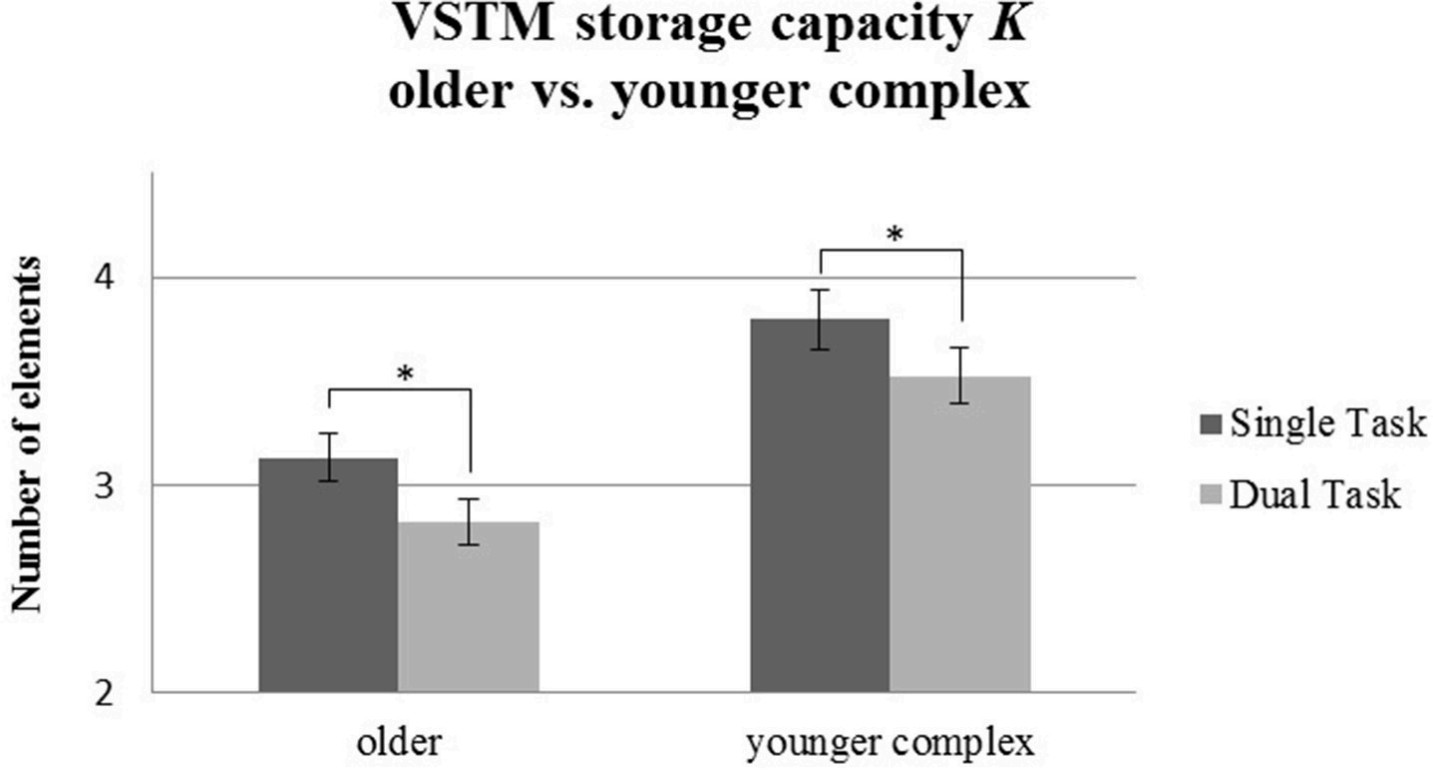
VSTM capacity *K* measured in maximum number of recognized letters for the older group performing the simple tapping sequence vs. the younger group performing the complex tapping sequence. Error bars indicate standard errors of the mean. Significant differences are denoted by an asterisk (^*^).

For VSTM storage capacity *K* (see Table 2), we found significant main effects of Age Group [*F*_(1, 58)_ = 15.69, *p* < 0.001, $\eta_{p}^{2}=$ 0.21] and Task Condition [*F*_(1, 58)_ = 35.87, *p* < 0.001, $\eta_{p}^{2}=$ 0.38], but no significant interaction [*F*_(1, 58)_ = 0.17, *p* = 0.68, $\eta_{p}^{2}=$ 0.003]. Thus, the older group showed a general reduction compared to the younger one in VSTM storage capacity *K*, and, across groups, dual task effects occurred. However, no indication was found for an enhanced dual task effect in VSTM storage capacity in the older group when a younger group had to perform a more challenging motor task. Figure 6 shows comparable reductions of VSTM storage capacity *K* for both age groups.

For parameter visual processing speed *C* (see Table 2), we did not find any significant effects [Age Group: *F*_(1, 58)_ = 0.03, *p* = 0.88; $\eta_{p}^{2}<$ 0.001; Task Condition: *F*_(1, 58)_ = 1.94, *p* = 0.17; $\eta_{p}^{2}=$ 0.03; Interaction: *F*_(1, 58)_ = 0.48, *p* = 0.49; $\eta_{p}^{2}=$ 0.008]. Thus, older and younger participants did not differ in visual processing speed, and none of the groups were affected by the secondary task.

We found a significant main effect for Age Group for visual threshold t_0_ (see Table 2) [*F*_(1, 58)_ = 17.42, *p* < 0.001, $\eta_{p}^{2}=$ 0.23], but no other significant effects [Task Condition: *F*_(1, 58)_ = 0.18, *p* = 0.68; $\eta_{p}^{2}=$ 0.003; Interaction: *F*_(1, 58)_ = 0.49, *p* = 0.49; $\eta_{p}^{2}=$ 0.008]. The visual threshold was significantly higher in the older group compared to the younger group performing the complex tapping sequence, but there were no indications for a difference in *t*_0_ between the single and dual task conditions in the younger or older groups.

### Goodness of fit

To test to what degree the empirical data obtained in the different experimental whole report conditions was explained by the TVA-based modeling, Goodness-of-fit measures were obtained. They showed that there was a close correspondence between the empirical data (mean accuracy scores) obtained in the different experimental conditions of the whole report and the values that would be predicted based on the TVA parameter estimates. The average Pearson product-moment correlation coefficients are listed in Table 3. They show for each participant group, and very similarly in single and dual task conditions, that at least 96% of the variance in the observed data is explained by the TVA model parameters. Across all participants, the model explained at least 89% of the variance. For individual Goodness-of-fit measures see Table 4 in the Supplementary Materials.

**Table 3:** Correlations between observed and modeled data: Goodness-of-Fit values (Pearson-product-moment correlation *r*) for single and dual-task-conditions for all three groups.

	**Single Task**	**Dual Task uncorrected**	**Dual Task corrected**
Older: Mn/SD/Range	0.97/0.02/0.896–0.997	0.96/0.02/0.901–0.996	0.96/0.03/0.901–0.998
Younger Simple: Mn/SD/Range	0.98/0.02/0.907–0.996	0.98/0.01/0.944–0.998	0.98/0.01/0.944–0.998
Younger Complex: Mn/SD/Range	0.98/0.02/0.922–0.998	0.98/0.02/0.905–1.00	0.98/0.02/0.906–1.0

Mn: Mean; SD: standard deviation

## Discussion

This study was aimed at specifying which aspects of visual attention capacity are disproportionately affected in elderly individuals in motor-cognitive dual task situations. To that end, we investigated the influence of a concurrent tapping task on the performance of a visual attention task (whole report) in older and younger participants, whilst additionally modulating the difficulty of the motor task performed by the younger adults. TVA model-based fitting of whole report performance provided estimates of separate visual attention capacity parameters.

When older participants performed a simple tapping task concurrently with the visual attention task, their VSTM storage capacity declined. However, when younger participants performed the same simple tapping sequence under dual task conditions, attention capacity did not show any significant decrement. However, in another group of younger participants performing a more challenging tapping task under dual task conditions, their VSTM storage capacity declined significantly as well. Tapping accuracy—although generally at a lower level in the older group than in the younger group performing the simple tapping task—remained unaffected by the load incurred by the dual task.

A comparison between the older participants performing the simple tapping, and the younger participants performing the complex tapping task, revealed that the effect of an additional tapping task on VSTM storage capacity was equally pronounced in both groups, although older adults, overall, had lower VSTM storage capacity than younger participants.

Similar to McAvinue et al. (2012) we found that older participants had a lower VSTM storage capacity, a higher visual threshold and—at least numerically—a lower perceptual processing speed than younger participants. These results are typical of older adults with normal or corrected-to-normal eyesight (see also Habekost et al., 2013; Espeseth et al., 2014). The fact that we did not see significant differences in perceptual processing speed seems to be driven by high standard deviations.

Taken together, these results shed considerable light on the nature of motor-cognitive dual task interference: Firstly, concurrent performance of a motor task seems to affect visual attention capacity quite selectively by way of reducing VSTM storage capacity. It was especially the number of items that could be maintained within VSTM that declined under dual task conditions. This was true both for older subjects performing the simple tapping, and for younger subjects performing the more complex tapping task. The remaining parameters obtained from TVA-based fitting were not significantly affected. That is, the perceptual threshold and the visual processing rate did not decline under dual-task compared to single-task conditions in any age group.

Secondly, the effect of the motor task on VSTM storage capacity appears to be more pronounced in older participants. Whilst the simple tapping sequence put only a minor demand on younger participants, this same task caused considerable dual task effects in the older adults. The VSTM decrement found in these older participants more or less equaled the decline revealed in younger adults performing the more complex tapping task. The aging effect thus seems to reflect the fact that a simple motor task is more challenging for older participants. In other words, even a simple motor program consisting of a sequence of concurrent finger tapping significantly decreased VSTM storage capacity in older adults, an effect which was only present to the same extent in younger adults when they performed a more complex motor task. Overall, the results of this study support capacity sharing accounts of dual tasking (e.g., Navon and Miller, 2002), implicating the VSTM storage capacity as being the limiting attentional capacity which is shared across the two tasks. Thus, as long as the capacity limits of the VSTM are not reached, the performance of both tasks remains unaffected. However, when the task demands exceed the limits of this capacity, such as when the task demands are increased, then the performance on the tasks is reduced.

In sum, our results show that the age-specific effects of motor-cognitive dual task interference are based on a stronger decline of VSTM storage capacity.

Our results are largely consistent with recent data presented by Künstler et al. (2017) who used the same method in a group of middle to higher aged subjects and combined the whole report task with the simple tapping task. In this study, a decrement of both VSTM storage capacity and processing rate was found under dual task conditions. The effect was more pronounced for VSTM, however, and a direct investigation of which parameter more strongly reflects the dual task related decline was not possible in this study. In line with these results, we found a clear decline of VSTM storage capacity in older subjects and in younger subjects performing a more complex tapping task, while the effects on processing rate were much weaker, and non-significant. Moreover, we were able to show that the age-related decline of attention capacity under motor-cognitive dual-task conditions is selectively reflected by parameter VSTM.

An important result of the Künstler et al. (2017) study was the demonstration that the performance of the whole report task, which was used to assess visual attention capacity, was qualitatively comparable under both single and dual task conditions. This was shown, for instance, by the fact that goodness-of-fit measures were comparable under both conditions. In this way, the valid applicability of the TVA-model—which assumes parameter estimates to remain constant across the task—under both single and dual task conditions was proven. Consequently, a conjecture that the whole report task would be performed in a non-continuous manner under dual task conditions (for example by switching attention between the two tasks) was not supported. Analogously, comparable goodness-of-fit measures across the single and the dual task conditions were obtained also in the present study. This in turn corroborates that participants performed both tasks simultaneously and continuously, as evidenced by the high correlations between the observed and the predicted data, also obtained in the present study. Thus, in congruence with the previous study, we would suggest that the results of the present study indicate that both tasks were executed simultaneously and in a qualitatively similar, although quantitatively less efficient way under the dual task as compared to single task condition.

The results of the present study are in line with earlier studies showing that motor-cognitive dual task interference is increased in aging (Kramer and Larish, 1996; Verhaeghen et al., 2002, 2003; Woollacott and Shumway-Cook, 2002; Boisgontier et al., 2013; Schaefer, 2014). They are also congruent with other studies which have indicated that increased task demands are linked with decreased spatial awareness during dual tasking (Lisi et al., 2015).

However, by referring to an explicit theoretical framework modeling attentional processing capacity, it was possible for the present study to specifically attribute the capacity limitation to the constraint in VSTM storage capacity.

To explain these findings, in the previous study (Künstler et al., 2017) we proposed that, when it comes to dual task situations, the VSTM represents a stage of response selection, at which verbal output is required in the whole report task, whilst simultaneously preparing the finger movement output for the tapping task. A similar view was proposed by Klapp (1976) who considered short-term memory as a stage of motor-response programming where response commands are temporarily stored. Under motor-cognitive dual-task conditions, when several response commands have to be maintained in parallel, the probability of interference at this stage is increased by cross-talk effects, resulting in a performance decline. Due to the fact that aging is associated with an overall decline of VSTM storage capacity, the reliability of maintained representations would be reduced in this group, giving rise to an even higher probability of interference (Jonides et al., 2008).

Of course, these assumptions are speculative and need to be investigated in future studies. However, they are in line with both a resource sharing perspective on short-term memory (Franconeri et al., 2013), as well as with the view that processing capacity limitations are mainly dependent on interference control and inhibition (Kane and Engle, 2002), which appears to be significantly reduced in older subjects (Mccabe et al., 2005).

It could be argued that our results might best be accounted for within Baddeley's multicomponent working memory model (see Baddeley, 2012, for a recent review). According to this view, motor-kinetic information from the finger tapping task and visual information from the whole report task would both be represented within the same slave system, namely the visuospatial sketch pad (VSSP). Doing both tasks in parallel would, therefore, increase the load on the VSSP compared to when each of the tasks is performed separately. A possible decrease in VSSP during aging (e.g., Kessels et al., 2010) would then mean that older participants have a higher load on modality specific resources than younger participants, while a more complex tapping pattern would mean a higher load even for younger participants. We consider such an explanation as less likely, for the following reasons. First, there is of course strong evidence that observed kinesthetic movement information (Baddeley, 2012) mentions gestures and dance as examples) is represented within the viewer's sketchpad. However, whether this is also true for motor programs representing sequential finger movements that are not directly observed remains equivocal. Moreover, Logie's seminal work (Logie, 1995) has shown that the VSSP itself can be subdivided into a visual and a spatial subsystem, with movement related information only tapping into the latter. This would be inconsistent with the assumption of a modality specific interference within the VSSP. In line with this assumption, recent ERP data of Katus and Eimer (2018) implies that tactile and visual working memory representations are distinct, i.e., modality-specific, and are not transferable across different sensory modalities

In conclusion, our results indicate that tasks are processed in parallel under conditions of motor-cognitive dual tasking, and that VSTM storage capacity is a core function involved in the dual task decrement, which is particularly exacerbated during aging. Whilst younger adults only show difficulties when the complexity of the secondary task is increased, older adults already show qualitatively similar decrements in the VSTM capacity when performing a simple secondary motor task.

## Author contributions

EK, MP, HM, KF, and PB contributed to the design of the study. NN contributed the necessary programming of the experiments used in this study. EK and MP collected the data, analyzed the results, and wrote the manuscript. KF and PB both supervised the data analysis and the writing of the manuscript. OW, CK, PB, and KF contributed to the data discussion. OW, PB, and CK contributed to the funding application. EK and MP contributed equally as first authors, whilst PB and KF both contributed equally as senior authors.

### Conflict of interest statement

The authors declare that the research was conducted in the absence of any commercial or financial relationships that could be construed as a potential conflict of interest.

## Footnotes

^1^For this study, we only analyzed tapping accuracy as a measure for effects of the dual task situation on the motor task. For the interested reader, average tapping speed and standard deviations as well as individual values and the distribution of tapping speed can be found in the Supplementary Materials in Tables 1, 4 and Figures 5–7.

^2^MATLAB and Statistics Toolbox Release. (2012). The MathWorks, Inc., Natick.

^3^Possibly due to subjects' inappropriate guessing during letter report, or to inefficient masking, TVA-based modeling provided negative *t0* values in multiple cases. We handled this problem by calculating our analyzes in two alternative ways: first, based on the model fit providing negative t0 values; second, based on a model fit constraining the minimum t0 value to zero. Both analyses generally revealed the same effects and group interactions. The data are provided in the Supplementary Materials in Tables 2, 3 and 5.
