# Supplementary material for: Dual Task Effects on Visual Attention Capacity in Normal Aging
Source: Front Psychol. 2018 Sep 3;9:1564. doi: 10.3389/fpsyg.2018.01564 (PMC6129777; doi:10.3389/fpsyg.2018.01564)
Supplement: Supplementary file 1 [file Table_1.docx]

**Sociodemographic Score**

Due to changes in educational and occupational standards over the years, we created a sociodemographic score based on vocabulary (an estimate of crystallized intelligence), number of school years, and occupation (either intended or obtained), with a maximum of 3 points being awarded per criterion. Thus, it was possible to obtain a minimum score of 3 and a maximum score of 9 points.

For the vocabulary score, each participant obtained a score based on his or her performance on the MWT-B (Lehrl, 1999), a German test which provides an estimate of crystallized intelligence. This was allocated as follows: 1 point for those below average; 2 points for those with an average score; and 3 points for those with an above average score. What was deemed to be below average, average, or above average was based on the norms set out in the MWT-B handbook (Lehrl, 1999).

Again, participants obtained a score between 1 and 3 based on their secondary school qualifications. Those completing a qualification which required 9 years of schooling obtained 1 point; those who completed a qualification which necessitates 10 years of education were awarded 2 points; and those who had a qualification which required 12 school years were given 3 points.

Finally, participants were scored according to their occupation. 1 point was given to those participants with menial jobs which did not require any further training or education; 2 points were given to those whose occupation required further training; 3 points were awarded to those participants with occupations requiring a university degree. University students were automatically awarded 3 points, even if they had not as yet completed their degree.

Older adults had a mean score of 7.4, with a standard deviation of 1.3, and a range of 5 to 9 points. The adults in the younger simple group (one value missing due to a missing IQ value) had a mean sociodemographic score of 6.7, a standard deviation of 1.4, and a range of 4 to 9 points. The younger complex group on the other hand had a mean score of 7.2, a standard deviation of 1.1, and a range of 5 to 9 points. There was no significant difference between the younger simple group and the older adults group (younger simple: *Mdn* = 7; older: *Mdn* = 7.5; *U* = 319.0, *p* = .073, r² = .05), nor between the younger complex group and the older adults group (younger complex: *Mdn* = 7; older: *Mdn* = 7.5; *U* = 397.5, *p* = .424, r² = .01). Please see Table 2 for the means and standard deviations of the scores for each group.

**Younger group performing the simple tapping sequence vs. younger group performing the complex tapping sequence**

To explore the differences between a simple versus a more complex tapping sequence in younger participants – which should increase the difficulty of the task – a comparison was run between the two younger groups.

**Tapping**

The comparison of the younger simple and younger complex groups showed a significant main effect of Tapping Group [*F*(1, 56) = 14.82, *p* < .001; $\eta_{p}^{2}$ = .21], but no other significant effects [Task Condition: *F*(1, 56) = .01, *p* = .91; $\eta_{p}^{2}$ < .001; interaction: *F*(1, 56) = .006, *p* = .94; $\eta_{p}^{2}$ < .001]. While the higher tapping demands led to lower overall accuracy in the group performing the complex compared to the group performing the simple sequence type, there was no indication for any dual task effect in tapping throughout the groups.

**Whole Report**

For VSTM storage capacity *K*, there was no significant main effect of Tapping Group [*F*(1, 58) = .0051; *p* = .94, $\eta_{p}^{2}$ < .001]. There was a significant main effect of Task Condition [*F*(1, 58) = 14.13, *p* < .001, $\eta_{p}^{2}$ = .20] and a significant interaction between Task Condition and Tapping Group[*F*(1, 58) = 4.77, *p* = .03, $\eta_{p}^{2}$ = .08]. Pairwise post-hoc *t*-tests with Bonferroni-correction showed a significant dual task effect on VSTM storage capacity only in the group performing the complex tapping sequence [*t*(29) = 3.98, *p* < .001, d = 0.35], and not in the group performing the simple tapping sequence [*t*(29) = .83, *p* = .41, d = 0.06; see Supplementary Figure 1].


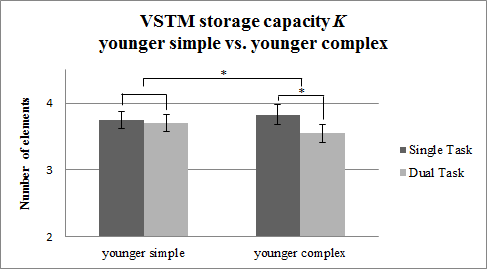


*Supplementary Figure 1:* VSTM capacity *K* measured in maximum number of recognized letters for the younger group performing the simple tapping sequence vs. the younger group performing the complex tapping sequence. Error bars indicate standard errors of the mean.

The respective ANOVA on processing speed *C* did not show any significant effects [Tapping Group: *F*(1, 58) = .24, *p* = .62; $\eta_{p}^{2}$ = .004; Task Condition: *F*(1, 58) = 1.55, *p* = .22; $\eta_{p}^{2}$ = .03; interaction: *F*(1, 58) = .28, *p* = .60; $\eta_{p}^{2}$ = .005]. Thus, visual processing speed was comparable across groups and was not affected by concurrent tapping.

For visual threshold *t_0_*, there were no significant main effects for Tapping Group [*F*(1, 58) = .05; *p* = .83, $\eta_{p}^{2}$ = .001] or Task Condition [*F*(1, 58) = .79; *p* = .38, $\eta_{p}^{2}$ = .01]and no significant interaction [*F*(1, 58) = .05; *p* = .83, $\eta_{p}^{2}$ = .001]. Thus, across different groups, task and complexity conditions, visual threshold *t_0_* remained rather constant.

These results indicate that when a complex motor program was performed as part of a dual task, the younger complex group experienced a significant reduction in the storage capacity of VSTM as compared to the younger adults performing the simple tapping sequence. This is in line with previous findings, which also showed that increased complexity can result in higher dual task decrements (Boisgontier et al., 2013). Processing speed and visual threshold were, however, unaffected. As higher tapping demands induced a specific decline in VSTM storage capacity only, this suggests that VSTM plays a role in supporting both the cognitive as well as the motor task in a dual tasking situation. If the overall cognitive load induced by dual tasking situation is relatively low, VSTM is able to successfully and accurately support both tasks simultaneously, with both tasks being processed in parallel. However, the time-point at which visual information starts to be processed, and the speed with which such information is processed was not affected by the complexity of the secondary task.

*Supplementary Table 1.* Tapping speed (seconds per tap) across all conditions and groups*.*

|  | **Single Task** | **Dual Task** |
| --- | --- | --- |
| **Older: Mn/ SD/ N** | .43/ .11/ 30 | .45/ .13/ 29 |
| **Younger Simple: Mn/ SD/ N** | .32/.11/29 | .29/.09/30 |
| **Younger Complex: Mn/ SD/ N** | .33/ .08/ 29 | .33/ .08/ 30 |

*Note.* Mn: Mean; SD: standard deviation; N = sample size

**Setting negative *t0*-values to 0**

Perhaps due to subjects’ inappropriate guessing during letter report, or to inefficient masking, TVA-based modeling provided negative *t0* values in multiple cases. We handled this problem by calculating our analyzes in two alternative ways: first, based on the model fit providing negative *t0* values; second, based on a model fit constraining the minimum *t0* value to zero. Both analyses generally revealed the same effects and group interactions. The data are provided in the Supplementary Tables 2, 3 and 5.

*Supplementary Table 2.* Results from repeated measures ANOVAs for TVA parameters *K* and *C* for all group comparisons (minimum *t0* = 0).

|  | **Younger simple vs. older simple** | | **Younger complex vs. older simple** | | **Younger simple vs. younger complex** | |
| --- | --- | --- | --- | --- | --- | --- |
|  | *K* | *C* | *K* | *C* | *K* | *C* |
| **Task Condition** |  |  |  |  |  |  |
| ***F*** | 18.24 | 6.58 | 39.23 | 2.07 | 16.05 | 1.02 |
| **df** | 1, 58 | 1, 58 | 1, 58 | 1, 58 | 1, 58 | 1, 58 |
| ***p-*value** | < .001** | .01* | < .001** | .16 | < .001** | .32 |
| $\eta_{p}^{2}$ | .24 | .10 | .40 | .04 | .22 | .02 |
| **Age Group/ Tapping Group** |  |  |  |  |  |  |
| ***F*** | 17.74 | 2.67 | 13.63 | 1.16 | .02 | .10 |
| **df** | 1, 58 | 1, 58 | 1, 58 | 1, 58 | 1, 58 | 1, 58 |
| ***p-*value** | < .001** | .11 | < .001** | .29 | .90 | .75 |
| $\eta_{p}^{2}$ | .23 | .04 | .19 | .02 | < .001 | .002 |
| **Interaction** |  |  |  |  |  |  |
| ***F*** | 9.42 | .03 | .07 | 2.72 | 4.86 | 1.44 |
| **df** | 1, 58 | 1, 58 | 1, 58 | 1, 58 | 1, 58 | 1, 58 |
| ***p-*value** | .003* | .87 | .79 | .11 | .03* | .24 |
| $\eta_{p}^{2}$ | .14 | < .001 | .001 | .05 | .08 | .02 |

*Note.* * *p* < .05; ** *p* < .001; df = degrees of freedom

Because of its non-normal distribution and thus a violation of assumptions that have to be met for the calculation of ANOVAs, non-parametric tests were used for the visual threshold *t_0_*. The results of these calculations can be found in Supplementary Table 3. Individual values for all TVA parameters (minimum *t0* = 0) are presented in Supplementary Table 5.

*Supplementary Table 3.* Results of Wilcoxon-Tests for all groups and of Mann-Whitney-*U*-Tests for all group comparisons for TVA parameter *t_0_* (minimum *t_0_* = 0).

| **Wilcoxon-Test** | | | | | | |
| --- | --- | --- | --- | --- | --- | --- |
|  | **Older simple** | | **Younger simple** | | **Younger complex** | |
| ***Z*** | -.024 | | -1.415 | | -.362 | |
| ***p*-value** | .98 | | .16 | | .72 | |
| ***r²*** | < .001 | | .07 | | .004 | |
| **Mann-Whitney-*U* Test** | | | | | | |
|  | **Older simple vs. younger simple** | | **Older simple vs. younger complex** | | **Younger simple vs. Younger complex** | |
|  | *single* | *dual* | *single* | *dual* | *single* | *dual* |
| ***Md*** | os = 10.00  ys = .44 | os = 11.35  ys = .17 | os = 10.00  yc = 1.59 | os = 11.35  yc = .81 | ys = .44  yc = 1.59 | ys = .17  yc = .81 |
| ***U*** | 173.0 | 189.0 | 152.0 | 189.0 | 443.5 | 438.0 |
| ***p*-value** | < .001** | < .001** | < .001** | < .001** | .92 | .85 |
| ***r²*** | .28 | .26 | .33 | .26 | < .001 | < .001 |

*Note.* Md = Median; os = older simple group; ys = younger simple group; yc = younger complex group; ** *p* < .001

*Supplementary figures 2 to 7*: Distribution of individual *K* parameter scores (S2-S4) and tapping speed (seconds per tap; S5-S7) for each group

S2.


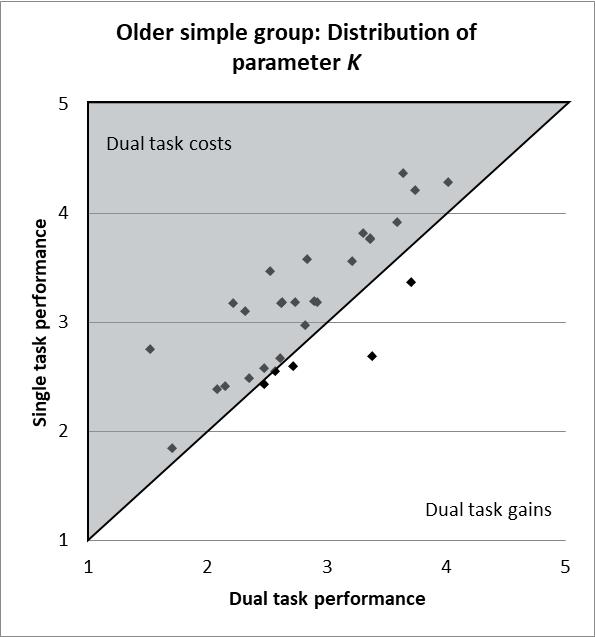


S3.


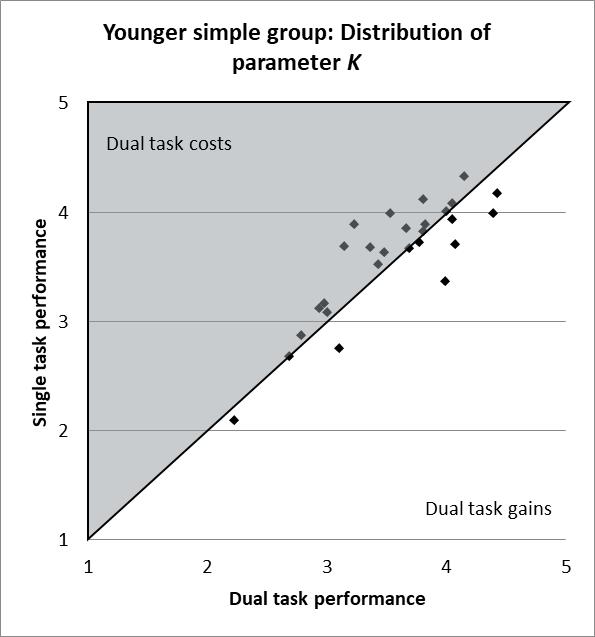


S4.


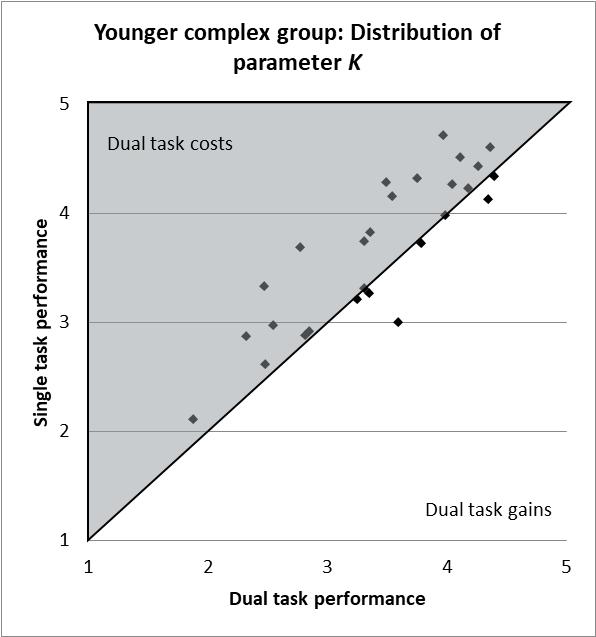


S5.


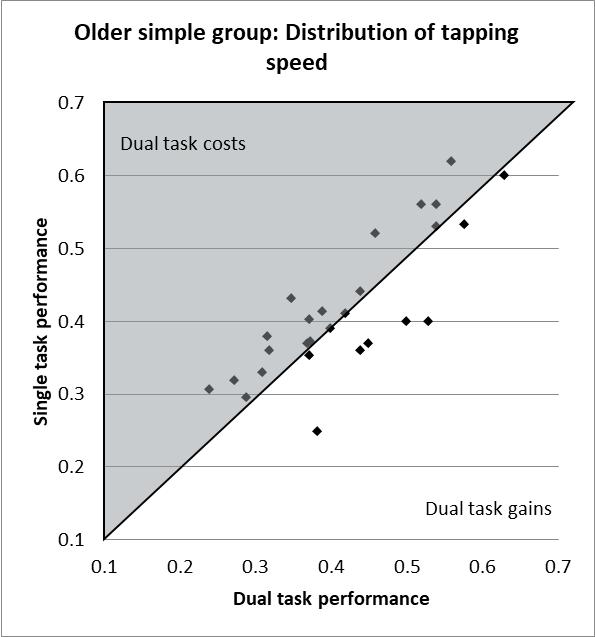


S6.


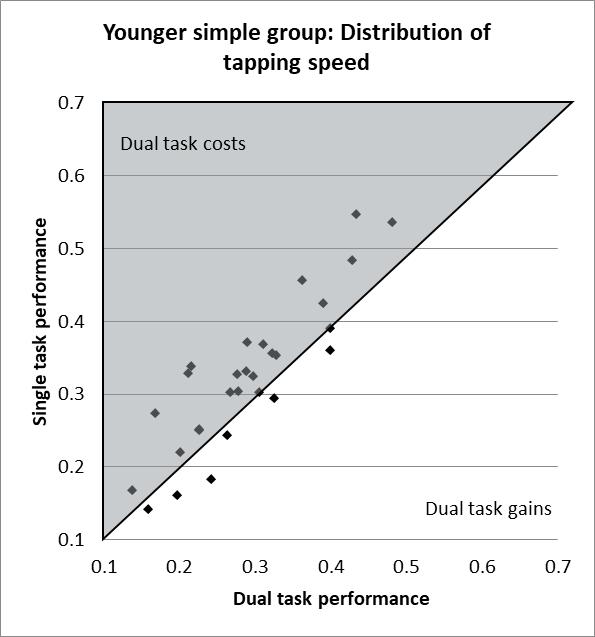


S7.


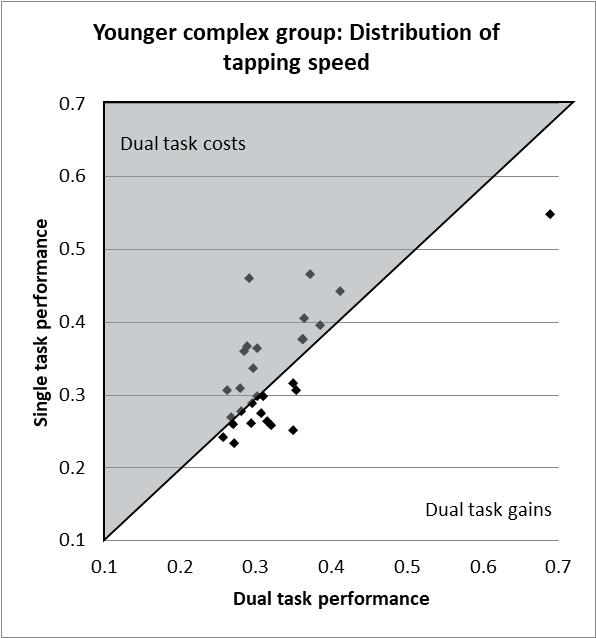


*Supplementary Table 4.* Individual values of Goodness-of-Fit (single, dual, with and without the exclusion of tapping errors) and of TVA parameters visual processing speed *C*, VSTM storage capacity *K* and visual threshold *t_0_* (single and dual task conditions) for each of the three groups.

| **ID** | **Goodness-of-fit** | | | **Parameter *C*** | | **Parameter *K*** | | **Parameter *t0*** | | **Tapping Accuracy** | | **Tapping Speed** | |
| --- | --- | --- | --- | --- | --- | --- | --- | --- | --- | --- | --- | --- | --- |
|  | Single | Dual uncorr. | Dual corr. | Single | Dual | Single | Dual | Single | Dual | Single | Dual | Single | Dual |
| YS01 | 0.989 | 0.992 | 0.952 | 23.35 | 35.16 | 4.08 | 4.06 | 5.04 | 10.00 | 98.97 | 99.42 | 0.30 | 0.28 |
| YS02 | 0.927 | 0.944 | 0.944 | 22.90 | 43.32 | 3.67 | 3.37 | -46.28 | -15.50 | 99.82 | 99.91 | 0.33 | 0.28 |
| YS03 | 0.992 | 0.974 | 0.974 | 90.19 | 70.87 | 3.89 | 3.83 | 14.31 | 8.80 | 100.00 | 99.87 | 0.24 | 0.26 |
| YS04 | 0.995 | 0.98 | 0.984 | 22.91 | 27.70 | 4.32 | 4.15 | 3.32 | 10.00 | 97.12 | 99.01 | 0.33 | 0.21 |
| YS05 | 0.996 | 0.984 | 0.987 | 33.74 | 19.09 | 3.99 | 3.54 | 18.12 | 8.61 | 97.37 | 98.14 | 0.17 | 0.14 |
| YS06 | 0.995 | 0.984 | 0.985 | 34.69 | 38.69 | 3.94 | 4.05 | .51 | -1.52 | 98.97 | 98.52 | 0.30 | 0.27 |
| YS07 | 0.988 | 0.965 | 0.967 | 29.09 | 17.20 | 3.69 | 3.15 | -4.15 | -22.17 | 100.00 | 99.01 | 0.33 | 0.29 |
| YS08 | 0.958 | 0.983 | 0.983 | 45.05 | 23.55 | 3.99 | 4.40 | -.04 | -5.77 | 99.38 | 99.60 | 0.22 | 0.20 |
| YS09 | 0.979 | 0.972 | 0.972 | 32.69 | 34.11 | 3.82 | 3.81 | 4.62 | -1.25 | 99.90 | 99.74 | 0.18 | 0.24 |
| YS10 | 0.991 | 0.996 | 0.996 | 27.18 | 33.86 | 3.63 | 3.49 | 2.76 | .35 | 100.00 | 99.47 | 0.55 | 0.43 |
| YS11 | 0.988 | 0.97 | 0.97 | 56.22 | 60.58 | 3.72 | 3.78 | 8.99 | 9.20 | -^1^ | 99.43 | -^1^ | 0.40 |
| YS12 | 0.923 | 0.998 | 0.998 | 36.93 | 22.16 | 2.75 | 3.11 | 10.00 | 4.31 | 97.34 | 95.78 | 0.35 | 0.33 |
| YS13 | 0.977 | 0.984 | 0.986 | 18.12 | 20.61 | 3.52 | 3.43 | -36.29 | -19.04 | 98.33 | 98.88 | 0.29 | 0.33 |
| YS14 | 0.98 | 0.994 | 0.995 | 25.85 | 36.54 | 3.89 | 3.23 | -3.50 | 7.67 | 100.00 | 97.31 | 0.37 | 0.29 |
| YS15 | 0.983 | 0.99 | 0.989 | 19.23 | 24.78 | 4.17 | 4.43 | -21.80 | -11.64 | 99.46 | 99.26 | 0.48 | 0.43 |
| YS16 | 0.984 | 0.994 | 0.994 | 17.00 | 15.52 | 2.68 | 2.69 | 14.48 | 8.13 | 99.82 | 99.82 | 0.32 | 0.30 |
| YS17 | 0.978 | 0.951 | 0.956 | 19.63 | 15.16 | 3.11 | 2.94 | -3.44 | -38.72 | 99.49 | 98.00 | 0.46 | 0.36 |
| YS18 | 0.991 | 0.958 | 0.954 | 17.69 | 16.59 | 2.09 | 2.23 | -1.01 | -11.09 | 97.08 | 97.92 | 0.34 | 0.22 |
| YS19 | 0.981 | 0.985 | 0.985 | 36.73 | 28.21 | 3.67 | 3.69 | -9.02 | -14.55 | 100.00 | 99.48 | 0.42 | 0.39 |
| YS20 | 0.992 | 0.976 | 0.977 | 37.08 | 38.21 | 5.24 | 4.97 | -1.78 | -1.04 | 98.79 | 99.05 | 0.36 | 0.32 |
| YS21 | 0.971 | 0.983 | 0.984 | 50.07 | 37.62 | 4.00 | 4.00 | 8.19 | 6.46 | 97.36 | 97.75 | 0.16 | 0.20 |
| YS22 | 0.985 | 0.982 | 0.982 | 13.93 | 10.15 | 2.87 | 2.79 | -22.35 | -26.16 | 100.00 | 100.00 | 0.27 | 0.17 |
| YS23 | 0.991 | 0.995 | 0.996 | 15.80 | 21.77 | 3.08 | 3.01 | .38 | -1.24 | 95.28 | 94.51 | 0.37 | 0.31 |
| YS24 | 0.938 | 0.991 | 0.991 | 35.46 | 40.26 | 4.86 | 5.10 | -24.23 | -3.46 | 95.14 | 99.05 | 0.30 | 0.31 |
| YS25 | 0.992 | 0.984 | 0.985 | 39.43 | 62.35 | 5.40 | 5.22 | -5.66 | 1.11 | 99.58 | 99.84 | 0.25 | 0.23 |
| YS26 | 0.983 | 0.977 | 0.968 | 27.78 | 25.98 | 3.16 | 2.98 | 10.00 | 8.89 | 98.00 | 98.95 | 0.25 | 0.23 |
| YS27 | 0.907 | 0.987 | 0.987 | 57.36 | 38.12 | 3.37 | 4.00 | 10.00 | 4.06 | 100.00 | 99.59 | 0.54 | 0.48 |
| YS28 | 0.989 | 0.978 | 0.975 | 42.21 | 27.00 | 3.70 | 4.08 | -7.26 | -23.92 | 97.40 | 99.72 | 0.14 | 0.16 |
| YS29 | 0.969 | 0.982 | 0.982 | 61.48 | 31.25 | 4.11 | 3.81 | 8.76 | 9.10 | 99.56 | 99.16 | 0.39 | 0.40 |
| YS30 | 0.981 | 0.957 | 0.956 | 40.36 | 25.43 | 3.85 | 3.67 | 12.40 | 10.00 | 99.46 | 99.12 | 0.14 | 0.16 |
| OS01 | 0.979 | 0.984 | 0.984 | 38.08 | 45.30 | 4.28 | 4.02 | 10.77 | 8.77 | 99.44 | 99.44 | 0.25 | 0.38 |
| OS02 | 0.949 | 0.921 | 0.921 | 48.37 | 33.47 | 2.68 | 3.38 | 4.89 | -9.97 | 99.41 | 100.00 | 0.53 | 0.58 |
| OS03 | 0.991 | 0.969 | 0.957 | 40.28 | 19.38 | 2.47 | 2.35 | 10.00 | -4.38 | 82.14 | 89.63 | 0.40 | 0.37 |
| OS04 | 0.952 | 0.979 | 0.979 | 42.77 | 43.31 | 3.90 | 3.59 | 2.51 | 5.16 | 98.97 | 99.07 | 0.31 | 0.24 |
| OS05 | 0.978 | 0.981 | 0.987 | 31.14 | 22.22 | 2.57 | 2.48 | 29.24 | 16.11 | 99.51 | 97.41 | 0.30 | 0.29 |
| OS06 | 0.991 | 0.968 | 0.967 | 11.04 | 20.37 | 2.74 | 1.53 | .48 | 4.80 | 97.11 | 94.49 | 0.44 | 0.44 |
| OS07 | 0.956 | 0.967 | 0.962 | 38.10 | 36.93 | 3.80 | 3.31 | 14.64 | 17.42 | 100.00 | 94.40 | 0.41 | 0.39 |
| OS08 | 0.98 | 0.98 | 0.975 | 17.23 | 13.91 | 1.83 | 1.71 | 33.93 | 55.02 | 97.37 | 92.48 | 0.35 | 0.37 |
| OS09 | 0.896 | 0.93 | 0.93 | 25.27 | 64.11 | 3.46 | 2.53 | -41.12 | -1.10 | 97.61 | 98.62 | 0.32 | 0.27 |
| OS10 | 0.985 | 0.959 | 0.959 | 20.85 | 21.20 | 2.96 | 2.82 | 10.00 | -6.73 | 99.71 | 98.82 | 0.53 | 0.70 |
| OS11 | 0.969 | 0.955 | 0.96 | 34.53 | 40.76 | 2.42 | 2.48 | 8.00 | 10.00 | 99.06 | 99.55 | 0.40 | 0.75 |
| OS12 | 0.993 | 0.974 | 0.964 | 42.32 | 42.75 | 2.59 | 2.72 | 6.67 | 7.22 | 96.62 | 93.00 | 0.40 | 0.50 |
| OS13 | 0.997 | 0.957 | 0.962 | 21.86 | 17.69 | 3.36 | 3.71 | 17.58 | 15.19 | 95.61 | 98.26 | 0.41 | 0.42 |
| OS14 | 0.976 | 0.953 | 0.959 | 13.68 | 15.37 | 2.37 | 2.09 | 7.84 | 13.31 | 98.49 | 89.28 | 0.36 | 0.44 |
| OS15 | 0.968 | 0.901 | 0.901 | 43.52 | 37.05 | 2.54 | 2.57 | 10.00 | 7.02 | 97.46 | 99.88 | 0.36 | 0.32 |
| OS16 | 0.981 | 0.981 | 0.981 | 32.24 | 22.60 | 3.75 | 3.37 | 15.03 | 15.94 | 95.47 | -^1^ | 0.39 | -^1^ |
| OS17 | 0.982 | 0.954 | 0.95 | 30.16 | 26.74 | 3.17 | 2.22 | 8.77 | 26.52 | 99.33 | 98.73 | 0.60 | 0.63 |
| OS18 | 0.993 | 0.977 | 0.977 | 35.77 | 25.07 | 3.17 | 2.74 | 18.28 | 18.21 | 98.92 | 98.42 | 0.74 | 0.55 |
| OS19 | 0.962 | 0.981 | 0.982 | 35.58 | 25.04 | 3.17 | 2.92 | 35.22 | 33.35 | 96.61 | 91.03 | 0.37 | 0.45 |
| OS20 | 0.966 | 0.994 | 0.997 | 29.13 | 26.17 | 3.09 | 2.32 | 20.00 | 20.00 | 99.41 | 96.82 | 0.62 | 0.56 |
| OS21 | 0.932 | 0.934 | 0.911 | 40.46 | 9.19 | 4.35 | 3.64 | 10.00 | 10.10 | 99.40 | 97.07 | 0.33 | 0.31 |
| OS22 | 0.944 | 0.983 | 0.985 | 31.97 | 17.55 | 2.66 | 2.62 | 20.00 | 20.00 | 98.13 | 91.07 | 0.53 | 0.54 |
| OS23 | 0.976 | 0.976 | 0.975 | 34.52 | 51.85 | 3.57 | 2.84 | 8.49 | 16.43 | 100.00 | 98.34 | 0.37 | 0.37 |
| OS24 | 0.997 | 0.982 | 0.998 | 21.44 | 16.94 | 3.16 | 2.62 | 31.14 | 37.11 | 99.74 | 95.00 | 0.52 | 0.46 |
| OS25 | 0.99 | 0.996 | 0.994 | 23.48 | 25.18 | 2.40 | 2.16 | 14.12 | 8.44 | 80.83 | 94.16 | 0.40 | 0.53 |
| OS26 | 0.989 | 0.949 | 0.949 | 28.33 | 23.02 | 3.18 | 2.90 | 16.17 | 7.55 | 100.00 | 99.78 | 0.56 | 0.54 |
| OS27 | 0.958 | 0.918 | 0.918 | 33.14 | 12.93 | 4.20 | 3.74 | 5.66 | -15.40 | 98.64 | 97.54 | 0.56 | 0.52 |
| OS28 | 0.974 | 0.98 | 0.974 | 40.70 | 31.53 | 3.75 | 3.37 | 5.40 | 8.40 | 99.76 | 95.42 | 0.43 | 0.35 |
| OS29 | 0.99 | 0.989 | 0.99 | 36.12 | 28.98 | 3.17 | 2.63 | 18.52 | 12.60 | 100.00 | 99.61 | 0.38 | 0.32 |
| OS30 | 0.955 | 0.976 | 0.965 | 29.52 | 42.79 | 3.54 | 3.22 | 5.28 | 13.55 | 100.00 | 99.66 | 0.37 | 0.37 |
| YC01 | 0.965 | 0.956 | 0.951 | 24.60 | 25.53 | 3.73 | 3.31 | -28.66 | -9.03 | 99.79 | 95.58 | 0.37 | 0.29 |
| YC02 | 0.98 | 0.994 | 0.993 | 32.82 | 42.63 | 4.22 | 4.18 | 7.08 | 4.18 | 98.93 | 97.42 | 0.37 | 0.36 |
| YC03 | 0.991 | 0.993 | 0.992 | 27.37 | 23.13 | 3.19 | 3.25 | 5.21 | 5.36 | 99.83 | 98.63 | 0.30 | 0.26 |
| YC04 | 0.998 | 0.983 | 0.986 | 32.68 | 24.02 | 3.30 | 3.31 | 3.76 | 2.99 | 92.56 | 91.61 | 0.26 | 0.32 |
| YC05 | 0.997 | 0.987 | 0.985 | 22.90 | 26.28 | 4.50 | 4.11 | -1.05 | 2.58 | 90.59 | 95.56 | 0.40 | 0.36 |
| YC06 | 0.986 | 1,000 | 1,000 | 18.06 | 18.96 | 3.71 | 3.79 | 10.00 | 12.16 | 97.51 | 99.29 | 0.55 | 0.69 |
| YC07 | 0.967 | 0.963 | 0.964 | 20.53 | 23.48 | 2.86 | 2.32 | 1.79 | 10.00 | 100.00 | 99.23 | 0.46 | 0.29 |
| YC08 | 0.994 | 0.982 | 0.988 | 58.49 | 44.37 | 4.59 | 4.37 | 8.07 | -5.70 | 97.28 | 95.82 | 0.33 | 0.30 |
| YC09 | 0.962 | 0.98 | 0.985 | 20.32 | 25.08 | 2.60 | 2.48 | 2.08 | 1.43 | 92.71 | 87.68 | 0.31 | 0.35 |
| YC10 | 0.976 | 0.996 | 0.995 | 48.43 | 26.00 | 4.14 | 3.54 | 4.52 | -4.42 | 97.02 | 94.81 | 0.26 | 0.27 |
| YC11 | 0.954 | 0.988 | 0.989 | 24.92 | 29.41 | 4.32 | 4.39 | 4.99 | .89 | 99.58 | 98.68 | 0.38 | 0.36 |
| YC12 | 0.988 | 0.98 | 0.978 | 17.66 | 19.93 | 2.87 | 2.81 | -8.38 | -16.84 | 98.58 | 95.90 | 0.28 | 0.28 |
| YC13 | 0.98 | 0.981 | 0.982 | 36.79 | 42.07 | 3.81 | 3.36 | -5.14 | 7.90 | -^1^ | 97.18 | - ^1^ | 0.30 |
| YC14 | 0.984 | 0.994 | 0.991 | 22.26 | 30.56 | 5.21 | 3.97 | -2.89 | -9.40 | 98.67 | 98.74 | 0.30 | 0.31 |
| YC15 | 0.964 | 0.981 | 0.98 | 22.99 | 21.54 | 3.67 | 2.77 | 7.46 | 7.12 | 98.24 | 99.43 | 0.44 | 0.41 |
| YC16 | 0.966 | 0.973 | 0.967 | 45.20 | 65.48 | 5.04 | 4.65 | -4.24 | 6.06 | 99.33 | 97.13 | 0.24 | 0.26 |
| YC17 | 0.985 | 0.993 | 0.993 | 23.66 | 24.29 | 4.71 | 3.97 | -11.65 | -.63 | 98.79 | 99.49 | 0.36 | 0.29 |
| YC18 | 0.952 | 0.974 | 0.974 | 38.36 | 43.32 | 3.97 | 3.99 | -6.15 | -8.59 | 99.17 | 98.12 | 0.30 | 0.30 |
| YC19 | 0.967 | 0.947 | 0.939 | 35.00 | 20.27 | 3.26 | 3.35 | -14.41 | -39.56 | 98.94 | 98.53 | 0.27 | 0.27 |
| YC20 | 0.997 | 0.999 | 0.999 | 25.50 | 24.57 | 4.11 | 4.35 | 15.01 | 8.17 | 89.54 | 96.17 | 0.26 | 0.29 |
| YC21 | 0.996 | 0.986 | 0.984 | 31.16 | 23.87 | 4.27 | 3.49 | -1.83 | -20.72 | 81.97 | 94.22 | 0.26 | 0.32 |
| YC22 | 0.922 | 0.905 | 0.906 | 15.44 | 16.68 | 4.31 | 3.75 | -62.34 | -49.99 | 100.00 | 99.60 | 0.46 | 0.37 |
| YC23 | 0.99 | 0.987 | 0.991 | 19.23 | 17.88 | 2.91 | 2.85 | 16.79 | -4.39 | 98.89 | 88.57 | 0.39 | 0.39 |
| YC24 | 0.977 | 0.977 | 0.976 | 46.77 | 53.24 | 4.25 | 4.05 | 2.94 | 1.57 | 97.20 | 97.07 | 0.29 | 0.30 |
| YC25 | 0.972 | 0.965 | 0.908 | 10.48 | 23.15 | 2.10 | 1.88 | 1.38 | 30.00 | 87.42 | 92.18 | 0.32 | 0.35 |
| YC26 | 0.955 | 0.979 | 0.977 | 19.52 | 19.21 | 2.99 | 3.60 | -10.25 | -25.08 | 99.18 | 98.36 | 0.36 | 0.30 |
| YC27 | 0.976 | 0.977 | 0.974 | 87.54 | 75.86 | 5.46 | 5.18 | -4.91 | -9.75 | 100.00 | 96.42 | 0.31 | 0.28 |
| YC28 | 0.991 | 0.985 | 0.983 | 33.91 | 21.15 | 2.96 | 2.55 | -1.99 | -8.77 | 93.10 | 98.79 | 0.23 | 0.27 |
| YC29 | 0.992 | 0.988 | 0.988 | 44.08 | 21.60 | 3.32 | 2.47 | 20.00 | .73 | 90.40 | 90.80 | 0.27 | 0.31 |
| YC30 | 0.988 | 0.974 | 0.973 | 28.90 | 30.94 | 4.41 | 4.26 | 10.89 | 17.35 | 94.64 | 96.53 | 0.25 | 0.35 |

*Note.* uncorr. = without the exclusion of tapping errors; corr. = tapping errors excluded; YS = younger simple group; OS = older simple group; YC = younger complex group; ^1^ missing value due to technical problems

*Supplementary Table 5.* Individual values of Goodness-of-Fit (single, dual, with and without the exclusion of tapping errors) and of TVA parameters visual processing speed *C*, VSTM storage capacity *K* and visual threshold *t_0_* (single and dual task conditions) for each of the three groups with the minimum value of *t_0_* fixed to 0.

| **ID** | **Goodness-of-fit** | | | **Parameter *C*** | | **Parameter *K*** | | **Parameter** *t_0_* | |
| --- | --- | --- | --- | --- | --- | --- | --- | --- | --- |
|  | Single | Dual uncorr. | Dual corr. | Single | Dual | Single | Dual | Single | Dual |
| YS01 | 0.989 | 0.992 | 0.952 | 23.35 | 35.16 | 4.08 | 4.06 | 5.04 | 10.00 |
| YS02 | 0.852 | 0.919 | 0.919 | 66.92 | 73.04 | 3.31 | 3.26 | .00 | .00 |
| YS03 | 0.992 | 0.974 | 0.974 | 90.19 | 70.87 | 3.89 | 3.83 | 14.31 | 8.80 |
| YS04 | 0.995 | 0.98 | 0.984 | 22.91 | 27.70 | 4.33 | 4.16 | 3.32 | 10.00 |
| YS05 | 0.996 | 0.984 | 0.987 | 33.74 | 19.09 | 3.99 | 3.54 | 18.12 | 8.61 |
| YS06 | 0.995 | 0.984 | 0.984 | 34.69 | 40.55 | 3.94 | 4.01 | .51 | .00 |
| YS07 | 0.986 | 0.915 | 0.967 | 31.97 | 25.93 | 3.65 | 3.02 | .00 | .00 |
| YS08 | 0.958 | 0.984 | 0.982 | 45.10 | 26.27 | 3.99 | 4.29 | .00 | .00 |
| YS09 | 0.979 | 0.971 | 0.972 | 32.69 | 35.19 | 3.82 | 3.82 | 4.62 | .00 |
| YS10 | 0.991 | 0.996 | 0.996 | 27.18 | 33.86 | 3.63 | 3.49 | 2.76 | .35 |
| YS11 | 0.988 | 0.97 | 0.97 | 56.22 | 60.58 | 3.72 | 3.78 | 8.99 | 9.20 |
| YS12 | 0.923 | 0.998 | 0.998 | 36.93 | 22.16 | 2.75 | 3.11 | 10.00 | 4.31 |
| YS13 | 0.917 | 0.956 | 0.955 | 34.70 | 30.27 | 3.32 | 3.31 | .00 | .00 |
| YS14 | 0.977 | 0.994 | 0.995 | 27.50 | 36.54 | 3.88 | 3.23 | .00 | 7.67 |
| YS15 | 0.971 | 0.98 | 0.979 | 26.07 | 30.18 | 4.12 | 4.38 | .00 | .00 |
| YS16 | 0.984 | 0.994 | 0.994 | 17.00 | 15.52 | 2.68 | 2.69 | 14.48 | 8.13 |
| YS17 | 0.978 | 0.913 | 0.913 | 20.91 | 28.36 | 3.11 | 2.83 | .00 | .00 |
| YS18 | 0.99 | 0.94 | 0.936 | 18.14 | 21.76 | 2.09 | 2.18 | .00 | .00 |
| YS19 | 0.975 | 0.981 | 0.981 | 45.54 | 41.75 | 3.63 | 3.53 | .00 | .00 |
| YS20 | 0.992 | 0.975 | 0.977 | 38.36 | 38.96 | 5.23 | 4.97 | .00 | .00 |
| YS21 | 0.971 | 0.983 | 0.984 | 50.07 | 37.62 | 4.00 | 4.00 | 8.19 | 6.46 |
| YS22 | 0.962 | 0.957 | 0.957 | 19.57 | 15.22 | 2.84 | 2.65 | .00 | .00 |
| YS23 | 0.991 | 0.995 | 0.996 | 15.80 | 22.37 | 3.08 | 3.01 | .38 | .00 |
| YS24 | 0.917 | 0.99 | 0.991 | 62.37 | 43.46 | 4.59 | 5.04 | .00 | .00 |
| YS25 | 0.991 | 0.984 | 0.985 | 43.72 | 62.35 | 5.35 | 5.22 | .00 | 1.11 |
| YS26 | 0.983 | 0.977 | 0.968 | 27.78 | 25.98 | 3.16 | 2.98 | 10.00 | 8.89 |
| YS27 | 0.907 | 0.987 | 0.987 | 57.36 | 38.12 | 3.35 | 4.00 | 10.00 | 4.06 |
| YS28 | 0.982 | 0.934 | 0.935 | 51.78 | 45.04 | 3.66 | 3.79 | .00 | .00 |
| YS29 | 0.969 | 0.982 | 0.982 | 61.48 | 31.25 | 4.11 | 3.81 | 8.76 | 9.10 |
| YS30 | 0.981 | 0.957 | 0.956 | 40.36 | 25.43 | 3.85 | 3.67 | 12.40 | 10.00 |
| OS01 | 0.979 | 0.984 | 0.984 | 38.08 | 45.30 | 4.28 | 4.02 | 10.77 | 8.77 |
| OS02 | 0.949 | 0.917 | 0.917 | 48.37 | 47.33 | 2.68 | 3.27 | 4.89 | .00 |
| OS03 | 0.991 | 0.968 | 0.959 | 40.28 | 22.03 | 2.47 | 2.33 | 10.00 | .00 |
| OS04 | 0.952 | 0.979 | 0.979 | 42.77 | 43.31 | 3.90 | 3.59 | 2.51 | 5.16 |
| OS05 | 0.978 | 0.981 | 0.987 | 31.14 | 22.22 | 2.57 | 2.48 | 29.24 | 16.11 |
| OS06 | 0.991 | 0.968 | 0.967 | 11.04 | 20.37 | 2.74 | 1.53 | .48 | 4.80 |
| OS07 | 0.956 | 0.967 | 0.962 | 38.10 | 36.93 | 3.80 | 3.31 | 14.64 | 17.42 |
| OS08 | 0.98 | 0.98 | 0.975 | 17.23 | 13.91 | 1.83 | 1.71 | 33.93 | 55.02 |
| OS09 | 0.831 | 0.923 | 0.927 | 70.64 | 67.41 | 3.19 | 2.53 | .00 | .00 |
| OS10 | 0.985 | 0.956 | 0.959 | 20.85 | 25.07 | 2.96 | 2.78 | 10.00 | .00 |
| OS11 | 0.969 | 0.955 | 0.96 | 34.53 | 40.76 | 2.42 | 2.48 | 8.00 | 10.00 |
| OS12 | 0.993 | 0.974 | 0.964 | 42.32 | 42.75 | 2.59 | 2.72 | 6.67 | 7.22 |
| OS13 | 0.997 | 0.957 | 0.962 | 21.86 | 17.69 | 3.36 | 3.71 | 17.58 | 15.19 |
| OS14 | 0.976 | 0.953 | 0.959 | 13.68 | 15.37 | 2.37 | 2.09 | 7.84 | 13.31 |
| OS15 | 0.968 | 0.901 | 0.901 | 43.52 | 37.05 | 2.54 | 2.57 | 10.00 | 7.02 |
| OS16 | 0.981 | 0.981 | 0.981 | 32.24 | 22.60 | 3.75 | 3.37 | 15.03 | 15.94 |
| OS17 | 0.982 | 0.954 | 0.95 | 30.16 | 26.74 | 3.17 | 2.22 | 8.77 | 26.52 |
| OS18 | 0.993 | 0.977 | 0.977 | 35.77 | 25.07 | 3.17 | 2.74 | 18.28 | 18.21 |
| OS19 | 0.962 | 0.981 | 0.982 | 35.58 | 25.04 | 3.17 | 2.92 | 35.22 | 33.35 |
| OS20 | 0.966 | 0.994 | 0.997 | 29.13 | 26.17 | 3.09 | 2.32 | 20.00 | 20.00 |
| OS21 | 0.932 | 0.934 | 0.911 | 40.46 | 9.19 | 4.35 | 3.64 | 10.00 | 10.10 |
| OS22 | 0.944 | 0.983 | 0.985 | 31.97 | 17.55 | 2.66 | 2.62 | 20.00 | 20.00 |
| OS23 | 0.976 | 0.976 | 0.975 | 34.52 | 51.85 | 3.57 | 2.84 | 8.49 | 16.43 |
| OS24 | 0.997 | 0.982 | 0.998 | 21.44 | 16.94 | 3.16 | 2.62 | 31.14 | 37.11 |
| OS25 | 0.99 | 0.996 | 0.994 | 23.48 | 25.18 | 2.40 | 2.16 | 14.12 | 8.44 |
| OS26 | 0.989 | 0.949 | 0.949 | 28.33 | 23.02 | 3.18 | 2.90 | 16.17 | 7.55 |
| OS27 | 0.958 | 0.918 | 0.893 | 33.14 | 12.88 | 4.20 | 3.97 | 5.66 | .00 |
| OS28 | 0.974 | 0.98 | 0.974 | 40.70 | 31.53 | 3.75 | 3.37 | 5.40 | 8.40 |
| OS29 | 0.99 | 0.989 | 0.99 | 36.12 | 28.98 | 3.17 | 2.63 | 18.52 | 12.60 |
| OS30 | 0.955 | 0.976 | 0.965 | 29.52 | 42.79 | 3.54 | 3.22 | 5.28 | 13.55 |
| YC01 | 0.925 | 0.955 | 0.947 | 46.17 | 31.59 | 3.52 | 3.26 | .00 | .00 |
| YC02 | 0.98 | 0.994 | 0.993 | 32.82 | 42.63 | 4.22 | 4.18 | 7.08 | 4.18 |
| YC03 | 0.991 | 0.993 | 0.992 | 27.37 | 23.13 | 3.19 | 3.25 | 5.21 | 5.36 |
| YC04 | 0.998 | 0.983 | 0.986 | 32.68 | 24.02 | 3.30 | 3.31 | 3.76 | 2.99 |
| YC05 | 0.996 | 0.987 | 0.985 | 23.29 | 26.28 | 4.51 | 4.11 | .00 | 2.58 |
| YC06 | 0.986 | 1,000 | 1,000 | 18.06 | 18.96 | 3.71 | 3.79 | 10.00 | 12.16 |
| YC07 | 0.967 | 0.963 | 0.964 | 20.53 | 23.48 | 2.86 | 2.32 | 1.79 | 10.00 |
| YC08 | 0.994 | 0.977 | 0.984 | 58.49 | 52.32 | 4.59 | 4.31 | 8.07 | .00 |
| YC09 | 0.962 | 0.98 | 0.985 | 20.32 | 25.08 | 2.60 | 2.48 | 2.08 | 1.43 |
| YC10 | 0.976 | 0.995 | 0.993 | 48.43 | 28.13 | 4.14 | 3.54 | 4.52 | .00 |
| YC11 | 0.954 | 0.988 | 0.989 | 24.92 | 29.41 | 4.32 | 4.39 | 4.99 | .89 |
| YC12 | 0.981 | 0.975 | 0.972 | 20.87 | 27.71 | 2.82 | 2.78 | .00 | .00 |
| YC13 | 0.977 | 0.981 | 0.982 | 41.90 | 42.07 | 3.79 | 3.36 | .00 | 7.90 |
| YC14 | 0.981 | 0.988 | 0.984 | 23.33 | 37.49 | 5.18 | 3.90 | .00 | .00 |
| YC15 | 0.964 | 0.981 | 0.98 | 22.99 | 21.54 | 3.67 | 2.77 | 7.46 | 7.12 |
| YC16 | 0.964 | 0.973 | 0.967 | 51.38 | 65.48 | 4.89 | 4.65 | .00 | 6.06 |
| YC17 | 0.978 | 0.993 | 0.993 | 28.53 | 24.56 | 4.61 | 3.97 | .00 | .00 |
| YC18 | 0.947 | 0.966 | 0.967 | 44.93 | 56.20 | 3.90 | 3.91 | .00 | .00 |
| YC19 | 0.941 | 0.842 | 0.848 | 51.79 | 54.19 | 3.19 | 2.92 | .00 | .00 |
| YC20 | 0.997 | 0.999 | 0.999 | 25.50 | 24.57 | 4.11 | 4.35 | 15.01 | 8.17 |
| YC21 | 0.995 | 0.956 | 0.961 | 32.28 | 36.13 | 4.26 | 3.39 | .00 | .00 |
| YC22 | 0.818 | 0.833 | 0.833 | 47.70 | 44.50 | 3.73 | 3.32 | .00 | .00 |
| YC23 | 0.99 | 0.988 | 0.991 | 19.23 | 19.49 | 2.91 | 2.83 | 16.79 | .00 |
| YC24 | 0.977 | 0.977 | 0.976 | 46.77 | 53.24 | 4.25 | 4.05 | 2.94 | 1.57 |
| YC25 | 0.972 | 0.965 | 0.908 | 10.48 | 23.15 | 2.10 | 1.88 | 1.38 | 30.00 |
| YC26 | 0.939 | 0.953 | 0.977 | 24.28 | 30.55 | 2.92 | 3.38 | .00 | .00 |
| YC27 | 0.977 | 0.971 | 0.974 | 103.34 | 101.33 | 5.41 | 5.11 | .00 | .00 |
| YC28 | 0.988 | 0.977 | 0.974 | 36.42 | 25.69 | 2.92 | 2.53 | .00 | .00 |
| YC29 | 0.992 | 0.988 | 0.988 | 44.08 | 21.60 | 3.32 | 2.47 | 20.00 | .73 |
| YC30 | 0.988 | 0.974 | 0.973 | 28.90 | 30.94 | 4.41 | 4.26 | 10.89 | 17.35 |

*Note.* uncorr. = without the exclusion of tapping errors; corr. = tapping errors excluded; YS = younger simple group; OS = older simple group; YC = younger complex group
